# Study on the Low-Damage Mechanism of Green ZrO_2_ Ceramics Under Ultrasonic Elliptical Vibration-Assisted Cutting

**DOI:** 10.3390/ma19173624

**Published:** 2026-08-26

**Authors:** Helin Chen, Fanshuo Jia, Qi Zhu, Yayun Liu, Chuanyang Wang

**Affiliations:** School of Mechanical and Electrical Engineering, Soochow University, Suzhou 215021, China; 15895437723@163.com (H.C.); 20235229047@stu.suda.edu.cn (F.J.); cc94028231@163.com (Q.Z.); cywang@suda.edu.cn (C.W.)

**Keywords:** green ceramics, ultrasonic vibration-assisted cutting, surface damage, wear resistance of cutting tool, discrete element simulation

## Abstract

Ultrasonic elliptical vibration was applied to the machining of green ZrO_2_ ceramics to investigate its effects on surface damage and tool wear. The experimental results were validated using discrete element simulations. Compared with conventional cutting, ultrasonic elliptical vibration-assisted cutting (UEVC) reduces surface roughness. The maximum reduction reaches 23.20% at a depth of cut of 0.8 mm and 25.39% at a cutting speed of 300 m/min. In addition, it reduces the average flank wear width by 11.5%. A substantial decrease in tangential force is observed at a depth of cut of 0.8 mm, an ultrasonic amplitude of 4 µm, and higher cutting speeds. Although the thickness of the surface compaction layer increases with cutting depth, UEVC reduces it by up to 59.33% at a depth of 0.8 mm. The PFC2D simulations predict lower subsurface residual stress magnitudes under UEVC than under CT. The chips generated during machining change from block-shaped chips under conventional cutting to powder-shaped chips under UEVC. This transformation facilitates chip removal. In addition, UEVC suppresses abrasive wear by minimizing scratching of the tool flank face from hard particles.

## 1. Introduction

At present, population aging, the increasing number of obese individuals, and the physiological decline associated with aging lead to a growing prevalence of knee joint diseases. Artificial knee joint replacement (TKA) is the main approach to correct deformities and improve joint function [1]. However, artificial knee joints suffer from severe wear, corrosion, and poor biocompatibility in service, which limit their service life and cost-effectiveness [2,3]. Because of their favorable tribological properties and biocompatibility, ceramic materials are considered promising alternatives for knee replacement applications [4].

However, the excellent physical and chemical properties of sintered ceramics also cause many machining defects [5,6]. These defects mainly appear as severe surface damage of the ceramic material [7] and rapid tool wear [8]. To address these issues, the processing of green ceramics has been introduced in recent years. Compared with machining sintered ceramics, green ceramic machining offers several advantages, including lower cost, higher machining efficiency, and reduced tool wear.

Green ceramics are unsintered ceramic compacts. Compared with sintered ceramics, their lower hardness and strength allow them to be shaped into desired forms by traditional mechanical processes. Green ceramic processing generally includes two main steps, namely green compact forming and subsequent machining [9,10]. Dry pressing [11], powder injection molding [12], and cold isostatic pressing [13] are used to prepare green ceramic compacts. These compacts exhibit sufficient strength to withstand traditional processing without fracture. For adequate machinability, the fracture strength of green ceramics should be at least 2 MPa [9].

During CNC milling of sintered alumina, the performance of CVD diamond-coated tools depends strongly on the cutting conditions, and coating delamination can occur [14]. Green-state machining provides an alternative. Both milling and laser ablation provide satisfactory dimensional accuracy for green alumina ceramics [15], while green ceramic machining allows a higher material removal rate than machining sintered ceramics. However, surface quality and tool wear remain important concerns in green ceramic machining. In the micromilling of green SiC and AlN ceramics, cutting parameters affect surface roughness and micro-tool wear [16]. Tool wear, in turn, significantly affects the surface roughness of both green and sintered ceramics [17].

UEVC is widely adopted to improve surface quality and reduce tool wear when processing hard and brittle materials [18,19], and it has attracted increasing research interest. UEVC is applied to the grinding of monocrystal sapphire, and the findings indicate that it reduces grinding force and surface roughness compared with conventional grinding [20]. Tool wear in ultrasonic vibration-assisted turning of SiC_f_/SiC ceramic matrix composites has also been investigated [21]. In green ceramic machining, Júnior et al. [22] apply unidirectional ultrasonic vibration along the depth-of-cut direction and report improvements in surface finish, cutting force, and flank wear.

However, the influence of an elliptical tool trajectory on the low-damage mechanisms of green ceramic machining remains insufficiently understood. To address this gap, the present study applies UEVC to the dry turning of cold-isostatically pressed green ZrO_2_ ceramics and combines cutting experiments with BPM simulations to investigate surface damage, compaction-layer formation, subsurface residual stress, cutting force, and tool wear. The low-damage mechanism is attributed to the intermittent cutting action induced by ultrasonic elliptical vibration. This action reduces tool–workpiece contact and cutting force. It also promotes the transformation from block-shaped chips to powder-shaped chips, facilitating chip evacuation. As a result, surface damage is further suppressed.

## 2. Experimental Method

### 2.1. Preparation of Green ZrO_2_ Ceramics

ZrO_2_ powder with a nominal particle size of approximately 200 nm is used in this study, and its chemical composition is listed in Table 1. The as-received powder contains 0.1 wt% premixed polyvinyl alcohol as a binder, and no additional binder is added during green compact preparation. The resulting green compacts have a measured bulk density of 3.08 g/cm^3^ and a porosity of 45.8%.

**Figure 1:**
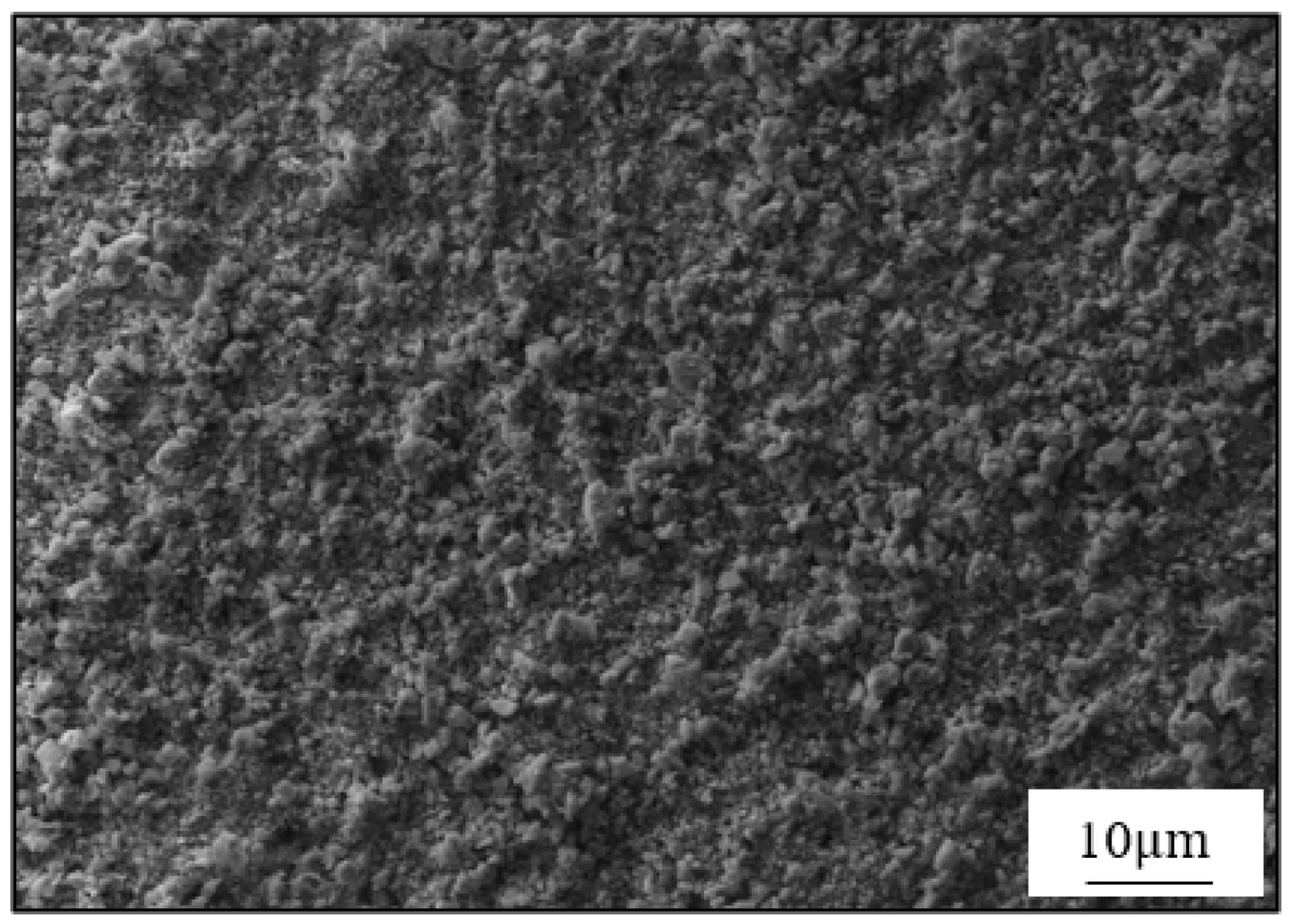
SEM image of the polished surface of green ZrO_2_ ceramics [23].

Green ZrO_2_ ceramic compacts are formed by cold isostatic pressing. The pressing parameters are a maximum pressure of 150 MPa, a holding time of 20 s, and a pressure relief/rise rate of 2.5 MPa/s. The dimensions of the pressed green ceramics are approximately Φ 60 mm × 200 mm. Figure 1 presents SEM images of the polished surface and cross-section of the green ZrO_2_ ceramics. Unlike sintered ceramics, the surface appears uniform and no grain boundaries exist between particle aggregates.

SCMT09T304 cemented-carbide inserts with a nose radius of 0.4 mm and honed cutting edges were used in the cutting experiments. The effective rake angle of the mounted insert is −5°. The geometric parameters of the cutting tool are listed in Table 2.

### 2.2. Ultrasonic Elliptical Vibration-Assisted Cutting of Green ZrO_2_ Ceramics

To test the cutting performance of ultrasonic vibration machining of green ceramics, the cutting tool material is chosen as cemented carbide, and the geometric properties of the cutting tool are shown in Table 2.

**Figure 2:**
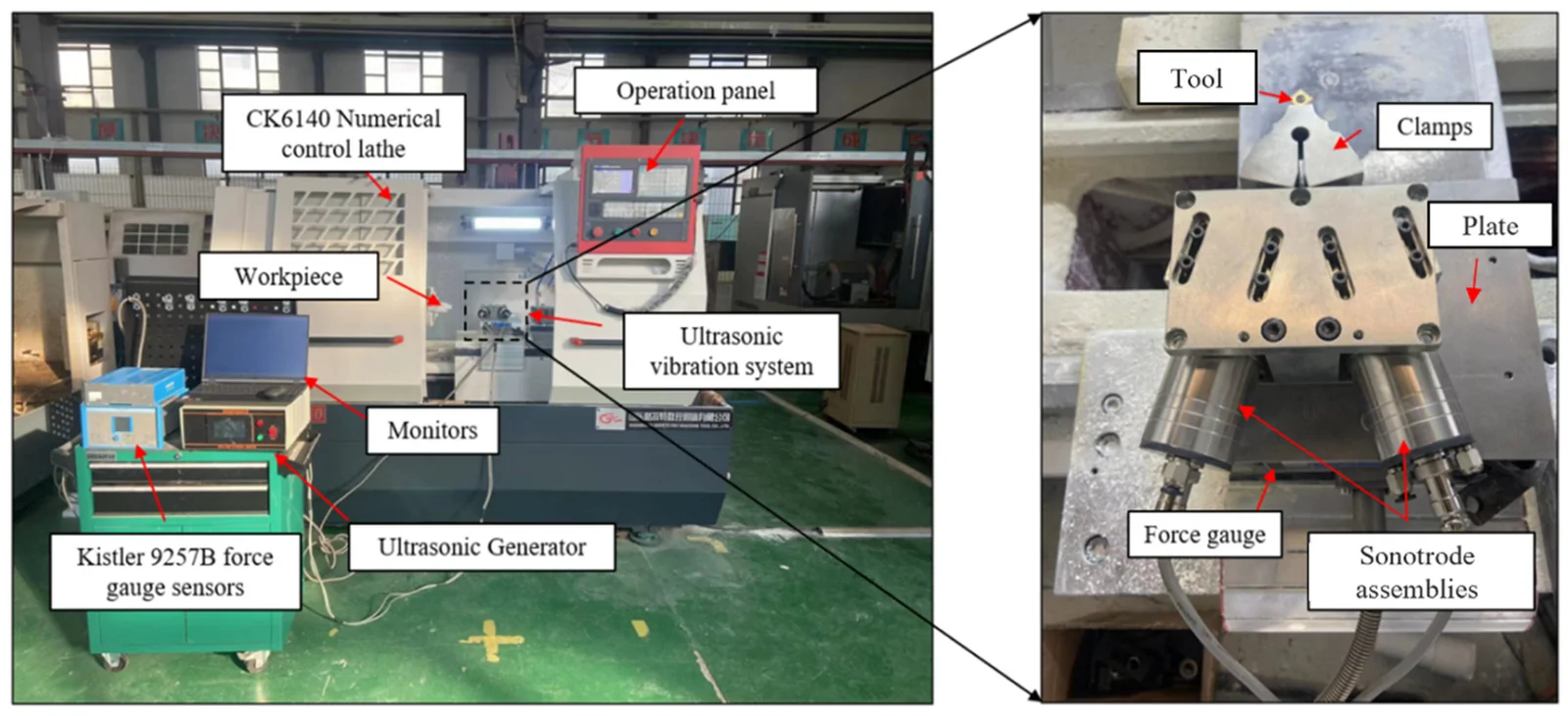
Ultrasonic vibration cutting test platform.

The tests are conducted on a CK6140 CNC lathe. As shown in Figure 2, the two inclined ultrasonic transducer–sonotrode assemblies are rigidly connected to an intermediate mounting plate positioned above the Kistler 9257B dynamometer (Kistler Instrumente AG, Winterthur, Switzerland). The intermediate plate is used to adjust the height of the cutting tool and to transmit the cutting forces acting on the tool to the dynamometer. The dynamometer is mounted on the moving platform of the lathe. During machining, the UEVC system applies micron-scale, high-frequency vibration to the cutting insert, and its main operating parameters are listed in Table 3. The ultrasonic generator supplies a sinusoidal electrical signal at 37.5 kHz. The piezoelectric transducers convert the electrical excitation into mechanical vibration through the inverse piezoelectric effect, while the sonotrodes amplify and transmit the vibration through the tool holder to the cutting insert.

A single-factor experimental design is adopted to investigate the effects of cutting depth, cutting speed, and ultrasonic amplitude. The feed rate is maintained at 0.015 mm/r throughout the experiments. For Tests 1–8, both CT (0 μm) and UEVC (4 μm) are performed under each condition. Tests 9–12 examine the effect of ultrasonic amplitude under UEVC at amplitudes of 2, 4, 6, and 8 μm, respectively. The specific test parameters are listed in Table 4. The machining results are used to analyze the surface-damage mechanisms of green ZrO_2_ ceramics under UEVC.

A separate tool-wear comparison is conducted at a cutting depth of 0.8 mm, a cutting speed of 100 m/min, and a feed rate of 0.015 mm/r. Both CT and UEVC tests cover the same cutting distance of 1500 m, corresponding to a net cutting time of 15 min. The vibration amplitude is 0 μm for CT and 6 μm for UEVC, and the UEVC frequency is 37.5 kHz. Except for the vibration conditions, the tool type, workpiece material, and all other cutting conditions are identical.

Surface roughness Ra is measured using a Kairda NDT130 portable stylus profilometer (Kairda Group Corporation, Beijing, China). The cutoff length and evaluation length are set to 2.5 and 12.5 mm, respectively. For each machined surface, three axial measurement paths at different circumferential positions are evaluated, and their average is taken as the Ra value for that test.

All cutting tests are performed in triplicate under each condition. Surface roughness, cutting force, and tool wear are reported as mean ± standard deviation based on the three repeated tests, and all error bars represent one standard deviation.

### 2.3. Ultrasonic Elliptical Vibration-Assisted Cutting of Green ZrO_2_ Ceramics by Discrete Element Method

**Figure 3:**
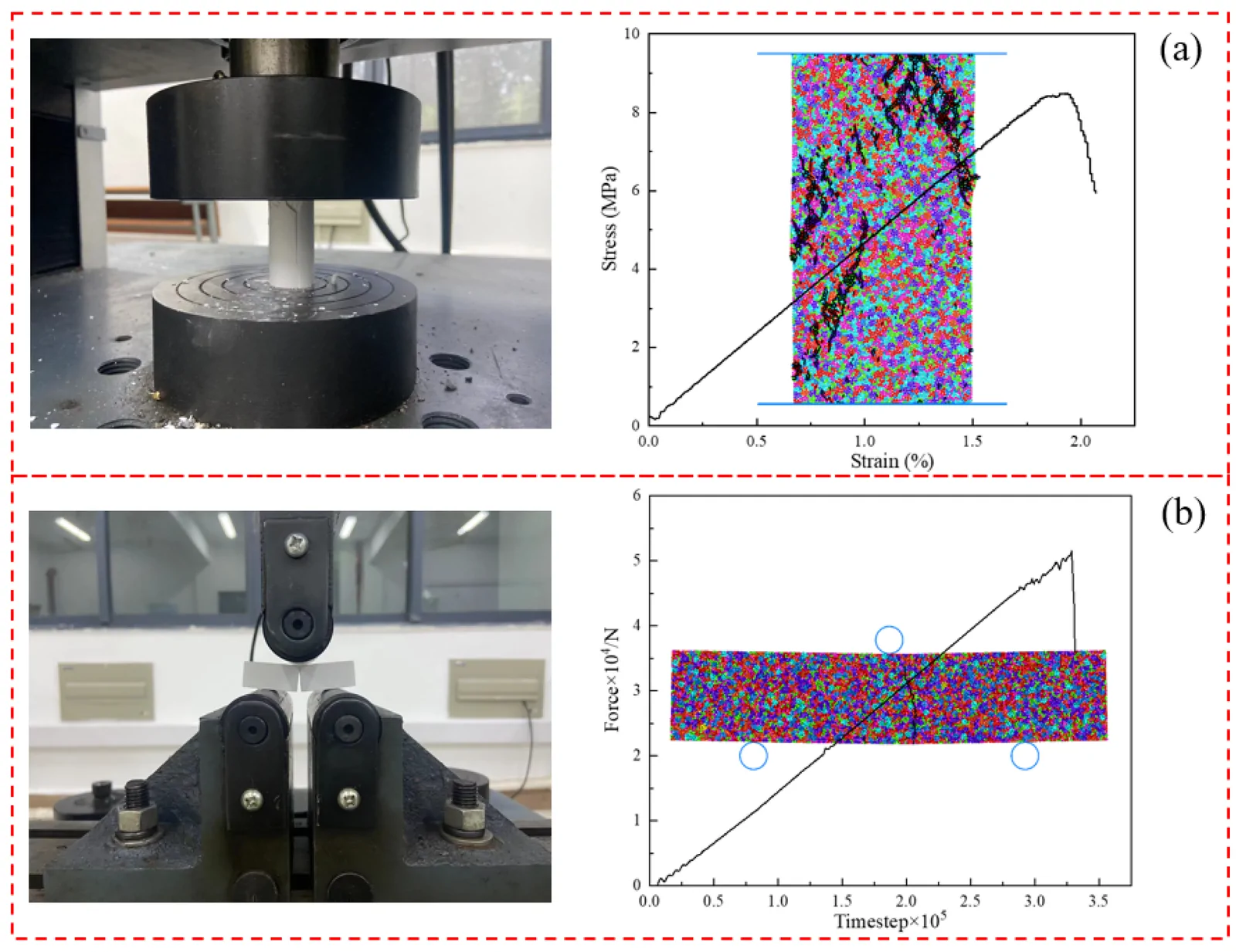
Discrete element calibration of green ZrO_2_ ceramics: (**a**) uniaxial compression test, (**b**) three-point bending test, in which the blue circles represent the upper loading wall and the two lower support walls [23].

This study adopts a previously established and calibrated two-dimensional BPM in PFC2D using the linear parallel-bond contact model [23]. Model particles with diameters ranging from 0.25 to 0.375 mm are randomly generated with an initial porosity of 0.10 and are grouped into clusters using FISH. Different parallel-bond strengths are assigned to intra- and inter-cluster contacts. For calibration (Figure 3), the uniaxial compression model measures 20 mm × 40 mm and contains 9249 particles, with the bottom wall fixed and the top wall moving downward at 1 mm/min. The three-point bending model measures 48 mm × 10 mm and contains 9152 particles, with a support span of 30 mm, fixed lower supports, and the upper loading wall moving downward at 0.1 mm/min. Particle stiffness, interparticle friction coefficient, local damping ratio, and cluster parallel-bond strengths are iteratively adjusted until the simulated macroscopic responses agree with the experimental results. The experimental/numerical values of flexural strength, compressive strength, and elastic modulus are 2.67/2.67 MPa, 8.40/8.49 MPa, and 465/458 MPa, respectively, yielding absolute relative errors of 0.00%, 1.07%, and 1.51%. The final calibrated parameters of the linear parallel-bond model are summarized in Table 5. The particle local damping coefficient is set to 0.7, and a bond installation gap of 1.2 × 10^−4^ model length units is used.

The calibrated parameters are applied to a cutting model measuring 60 mm × 30 mm that contains 8127 particles. The left, right, and bottom boundaries of the workpiece are fixed. The two-dimensional model represents a cross-section defined by the cutting direction and the surface-normal direction. Two connected rigid walls approximate the tool geometry, while their prescribed in-plane motion reproduces the elliptical tool trajectory projected onto this plane. The model enables qualitative analysis of tool–workpiece separation, bond breakage, and chip formation.

Subsurface residual stresses are evaluated using the measurement-circle method implemented in PFC2D. At each nominal subsurface depth from 0.1 to 0.8 mm at intervals of 0.1 mm, 50 measurement circles with a radius of 0.1 mm are evenly distributed beneath the machined surface. The stress magnitude and direction within each measurement circle are calculated to characterize the local stress state. The residual stress magnitudes obtained from the 50 measurement circles are arithmetically averaged to determine the mean residual stress magnitude at each depth. The results are reported as mean ± standard deviation, and the error bars represent the standard deviation among the 50 measurement-circle values.

## 3. Result

### 3.1. Tool Wear

**Figure 4:**
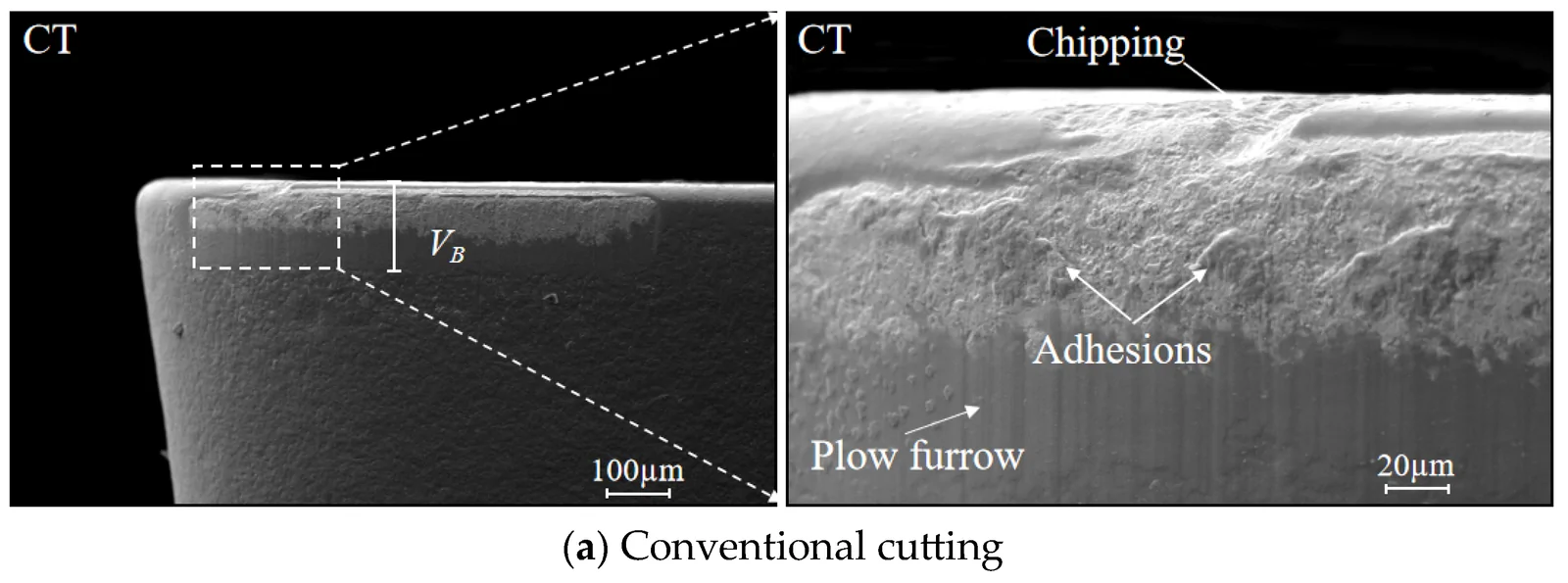

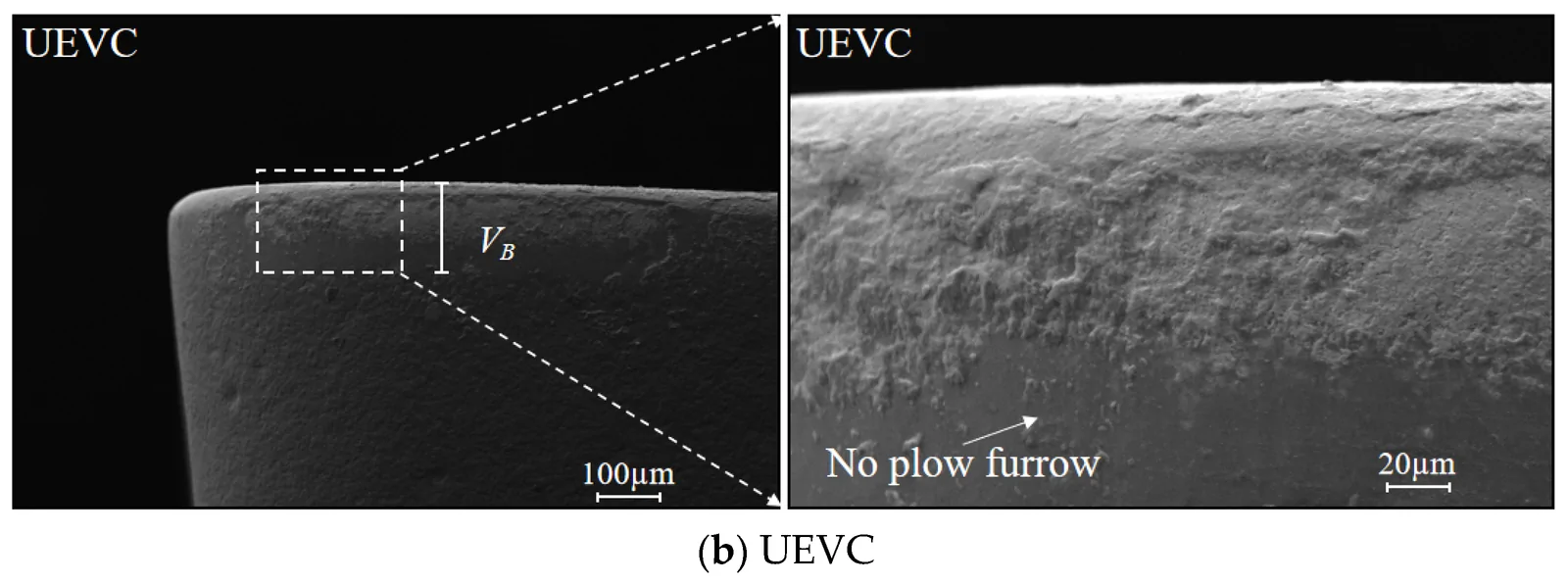
SEM image of tool wear on the flank face after machining green ZrO_2_ ceramics.

Figure 4 shows the flank wear of cutting tools after conventional cutting and UEVC. Figure 4a reveals that tool wear is more severe under conventional cutting. The enlarged view shows mechanical plowing furrows on the flank face, which is characteristic of abrasive wear caused by hard particles and chips. Adhered material also appears in the worn area. Moreover, defects are observed at the cutting edge, which result from chipping during machining. Figure 4b presents the flank wear morphology after UEVC. In the enlarged image, the plowing furrows generated by abrasive wear essentially disappear, and no chipping is observed. The average flank wear width VB is measured under both conditions. The average VB is about 156 ± 12 µm in conventional cutting and about 138 ± 8 µm in UEVC. The improvement in wear resistance reaches 11.5%.

### 3.2. Surface Morphology

#### 3.2.1. Effect of Depth of Cut on the Surface Morphology of Green ZrO_2_ Ceramics

**Figure 5:**
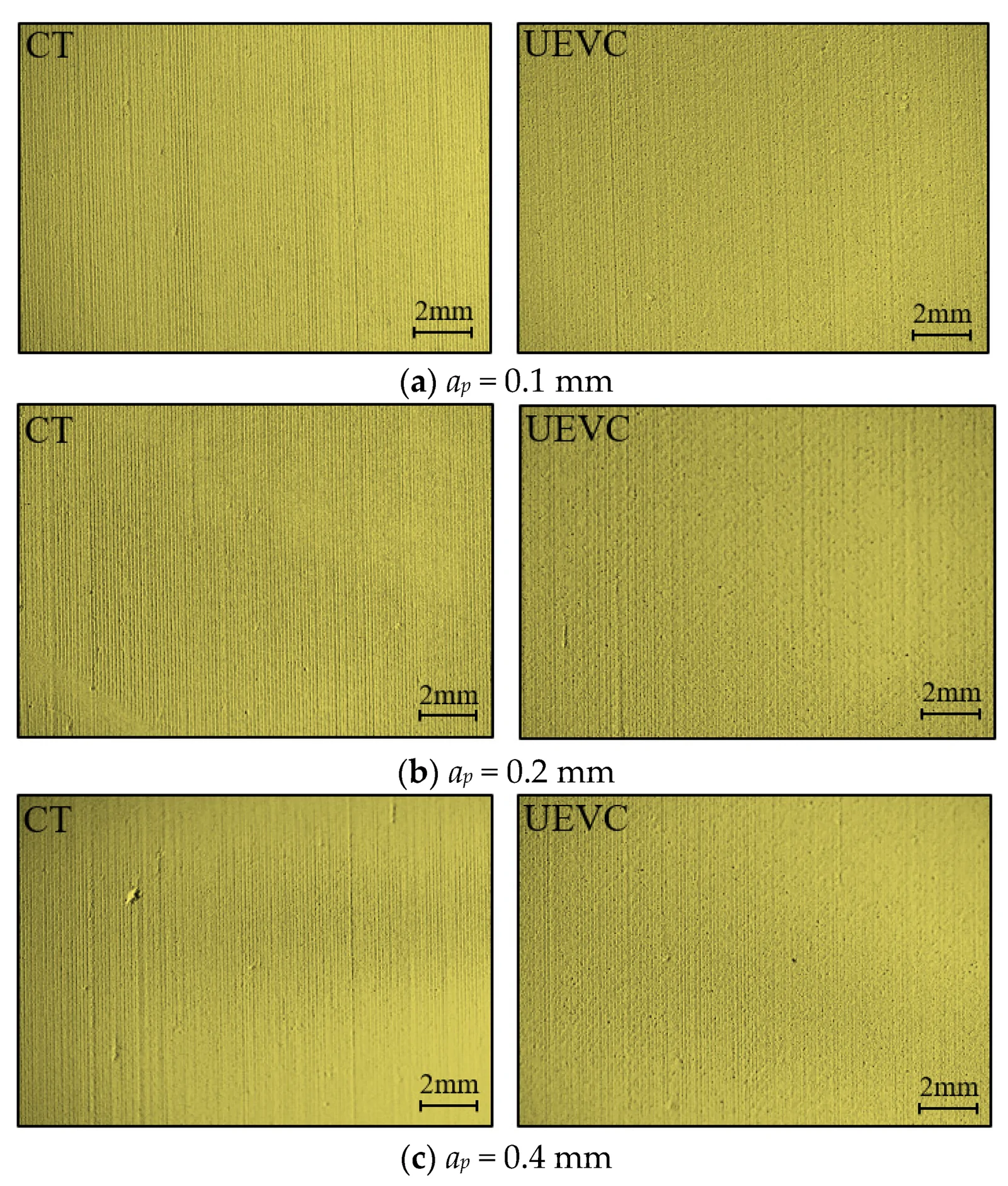

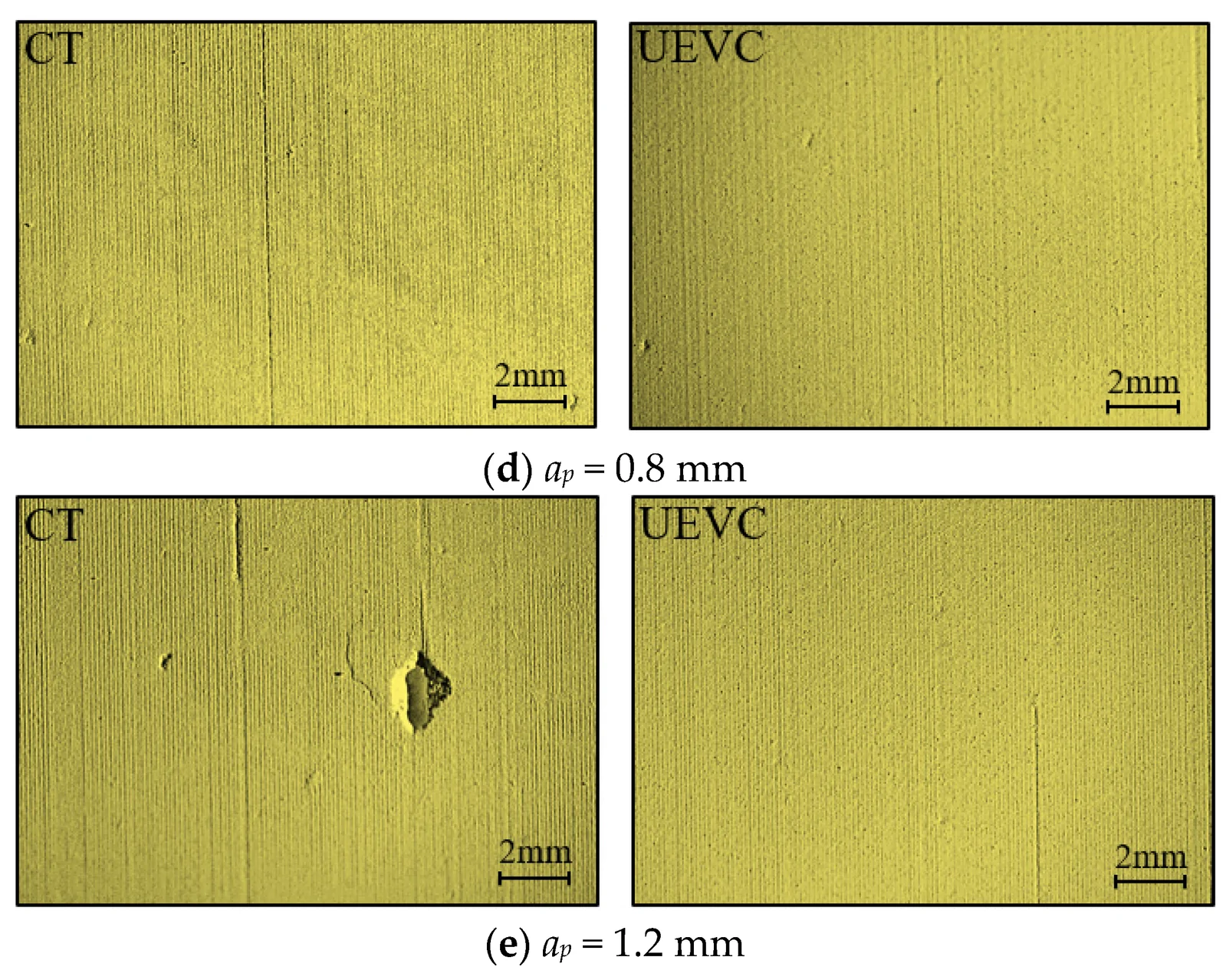
Surface morphology of green ZrO_2_ ceramics at different depths of cut.

Figure 5 presents the surface morphology of green ZrO_2_ ceramics machined at different depths of cut. CT denotes conventional cutting. As shown in Figure 5a,b, the machined surface is flat and free of obvious defects at small depths of cut. When the depth of cut increases to 0.8 mm, craters appear on the machined surface. Figure 5e shows that at a depth of cut of 1.2 mm, more pronounced defects such as craters are observed, and scratch marks caused by hard particles are clearly visible. In general, UEVC produces better surface quality than CT. As the depth of cut increases, the improvement in surface quality becomes more pronounced under UEVC, with fewer craters and tool marks.

This improvement is attributed to the elliptical motion of the tool induced by ultrasonic vibration. In one vibration cycle, the tool tip follows an elliptical trajectory, leading to periodic contact and separation between the tool and the workpiece. This intermittent cutting promotes chip removal and reduces secondary damage from chips on the machined surface [22].

**Figure 6:**
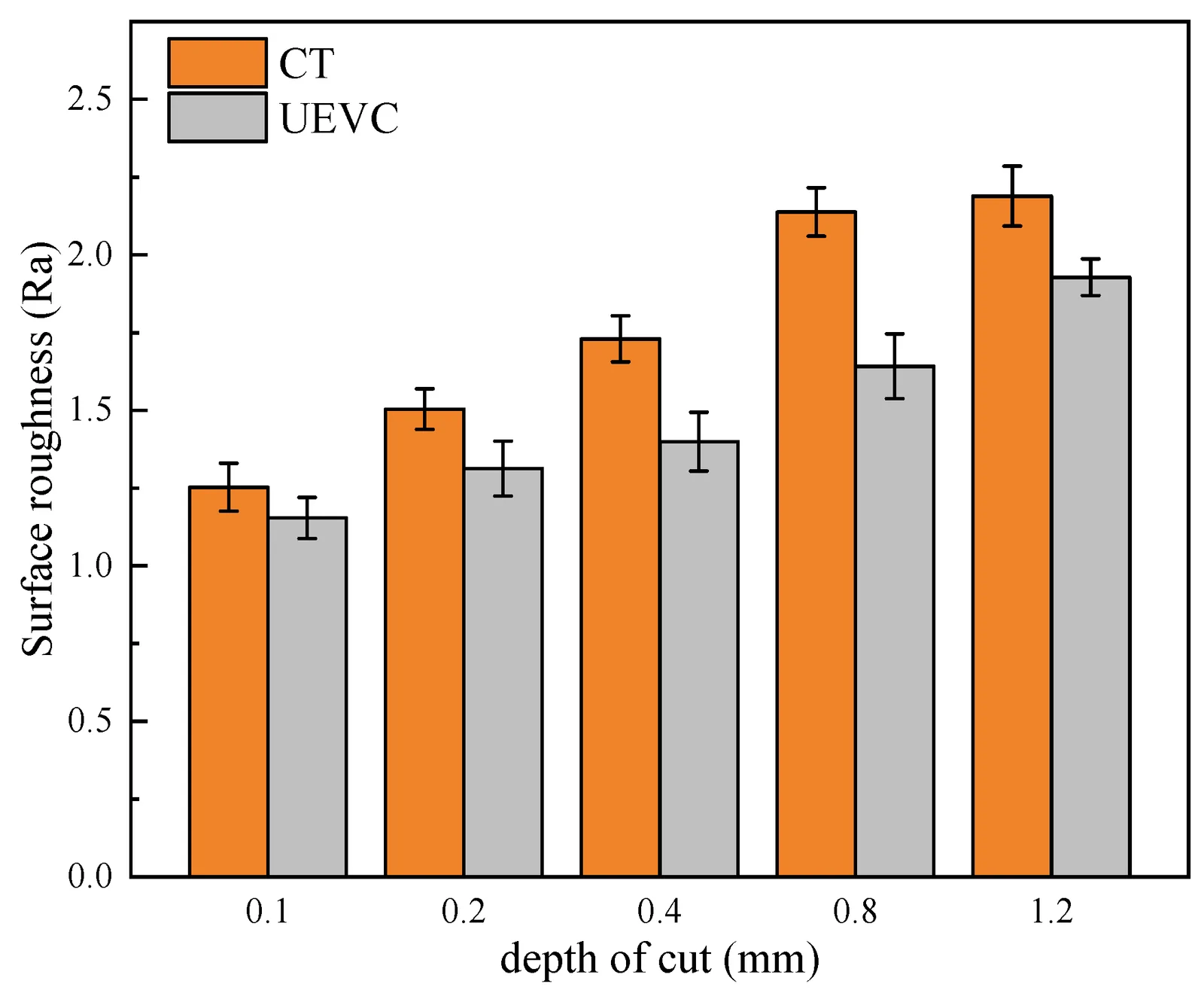
Effect of depth of cut on surface roughness of green ZrO_2_ ceramics [23].

Figure 6 shows the surface roughness of green ZrO_2_ ceramics under CT and UEVC. The surface roughness increases with the depth of cut under both cutting conditions. This trend is consistent with the surface damage morphology presented in Figure 5. At depths of cut of 0.1, 0.2, 0.4, 0.8, and 1.2 mm, the mean surface roughness values under UEVC are 7.8%, 12.7%, 19.1%, 23.2%, and 11.4% lower than those under CT, respectively. The largest measured difference occurs at a depth of cut of 0.8 mm.

#### 3.2.2. Effect of Cutting Speed on the Surface Morphology of Green ZrO_2_ Ceramics

**Figure 7:**
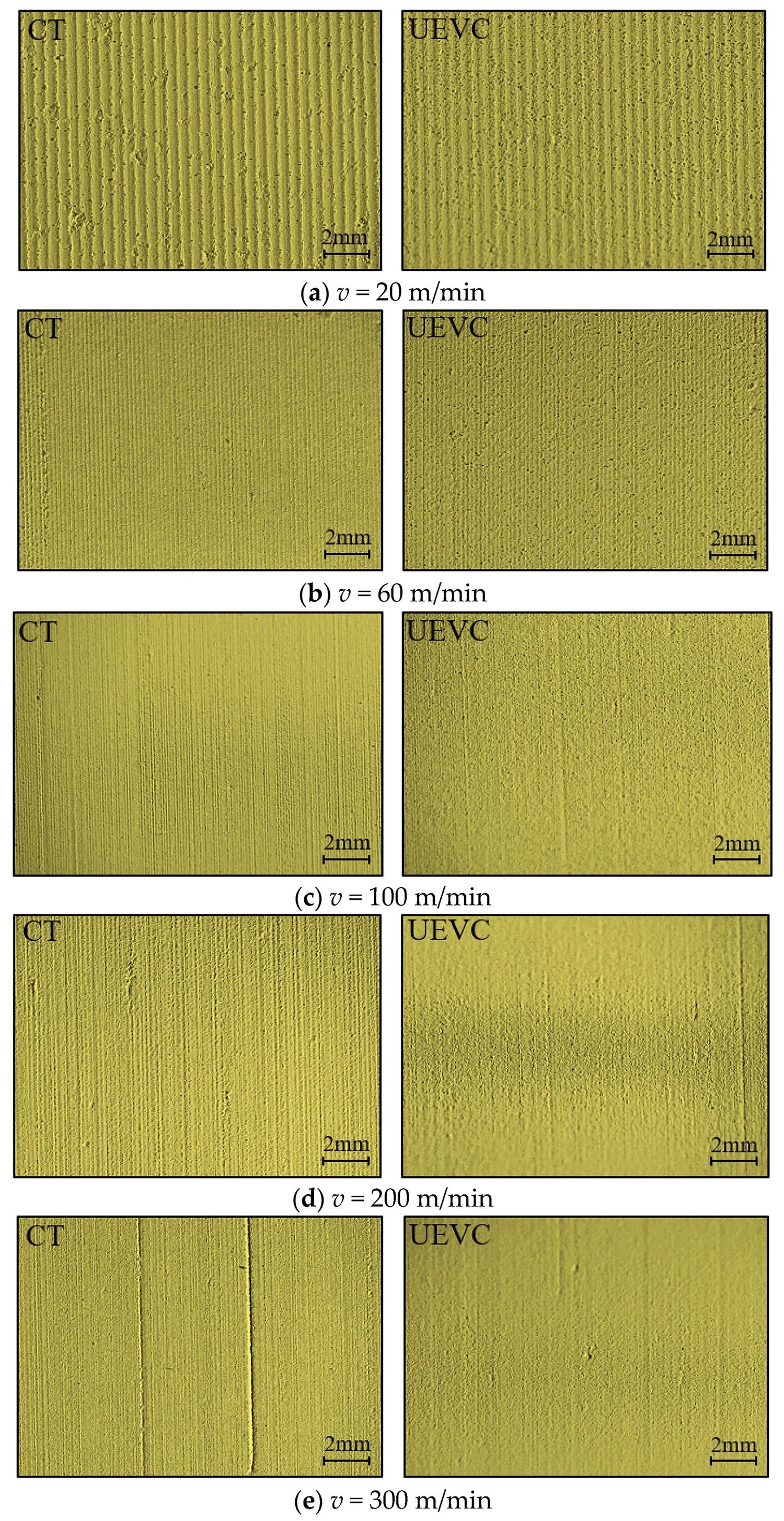
Surface profiles of green ZrO_2_ ceramics at different cutting speeds.

Figure 7 shows the machined surface topography of green ZrO_2_ ceramics under CT and UEVC at different cutting speeds. As shown in Figure 7a, at a cutting speed of 20 m/min, the machined surface quality is poor, with many craters. This indicates that the material is removed mainly as block-shaped chips. When the cutting speed increases to 100 m/min, the surface damage in the green ceramics decreases. The surface quality deteriorates at cutting speeds above 200 m/min. Tool marks become more obvious, and more pronounced defects appear in Figure 7d,e. At a cutting speed of 300 m/min, the machined surface quality is poor, and scratches caused by hard particles are clearly visible. UEVC reduces these scratches. Overall, UEVC of green ZrO_2_ ceramics improves the surface profile and reduces surface damage.

**Figure 8:**
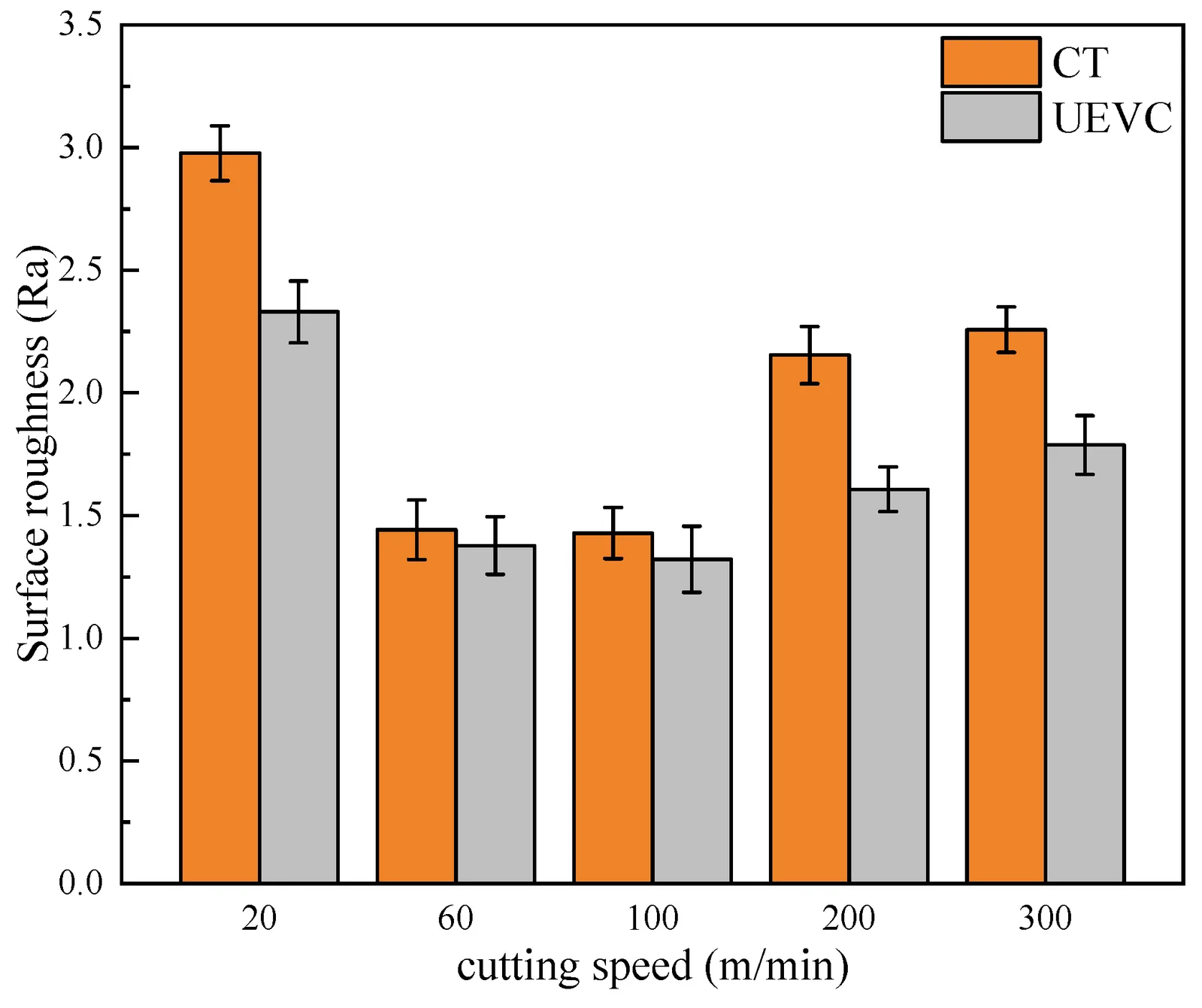
Effect of different cutting speeds on the surface roughness of green ZrO_2_ ceramics.

Figure 8 presents the surface roughness of workpieces machined under CT and UEVC at various cutting speeds. For green ZrO_2_ ceramics, the surface roughness reaches its maximum at a cutting speed of 20 m/min. As the cutting speed increases, the surface roughness initially decreases and then rises. The roughness values at 60 m/min and 100 m/min are comparable, which is consistent with the surface morphologies shown in Figure 7. Notably, UEVC consistently yields lower surface roughness than CT. Thus, ultrasonic vibration not only improves surface finish and reduces surface damage but also demonstrates that an appropriate cutting speed can further enhance surface quality.

#### 3.2.3. Effect of Amplitude on the Surface Morphology of Green ZrO_2_ Ceramics

**Figure 9:**
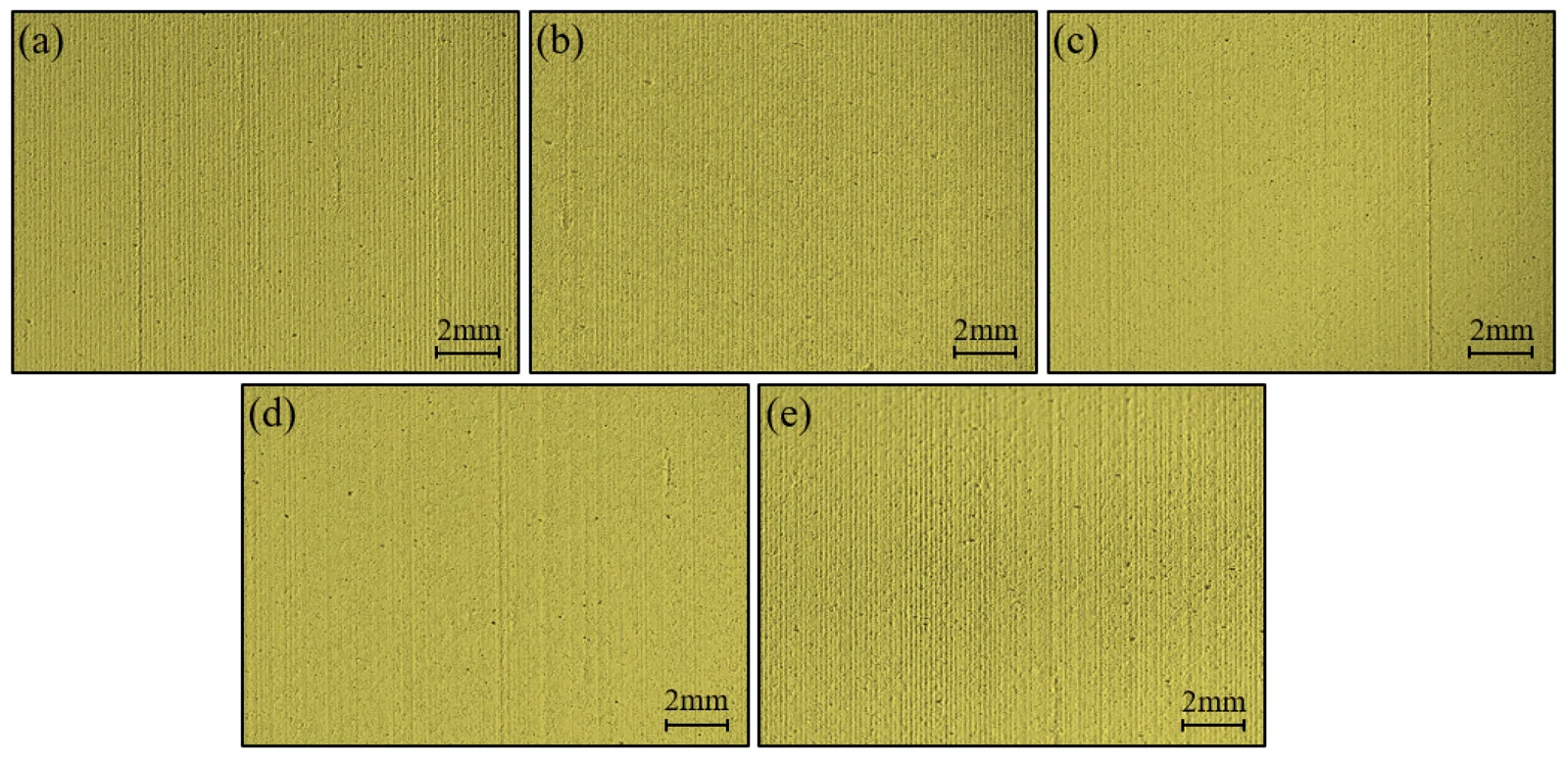
Surface morphology of green ZrO_2_ ceramics at different ultrasonic amplitudes: (**a**) 0 µm; (**b**) 2 µm; (**c**) 4 µm; (**d**) 6 µm; (**e**) 8 µm.

The effect of ultrasonic amplitude on the surface damage of green ZrO_2_ ceramics is investigated over a range from 0 to 8 µm. Figure 9 shows the surface morphology at different ultrasonic amplitudes. At an amplitude of 2 µm, the machined surface is flatter than that without ultrasonic vibration, and the tool marks are shallow. The lowest surface roughness within the tested amplitude range is obtained at 4 µm. At 6 µm, a small amount of hard particles remains on the surface. At 8 µm, the machined surface becomes rougher, and a large number of hard particle agglomerates adhere to the surface, as shown in Figure 9e. Therefore, as the ultrasonic amplitude increases, the surface quality first improves and then deteriorates. This trend is consistent with the surface morphologies shown in Figure 9.

**Figure 10:**
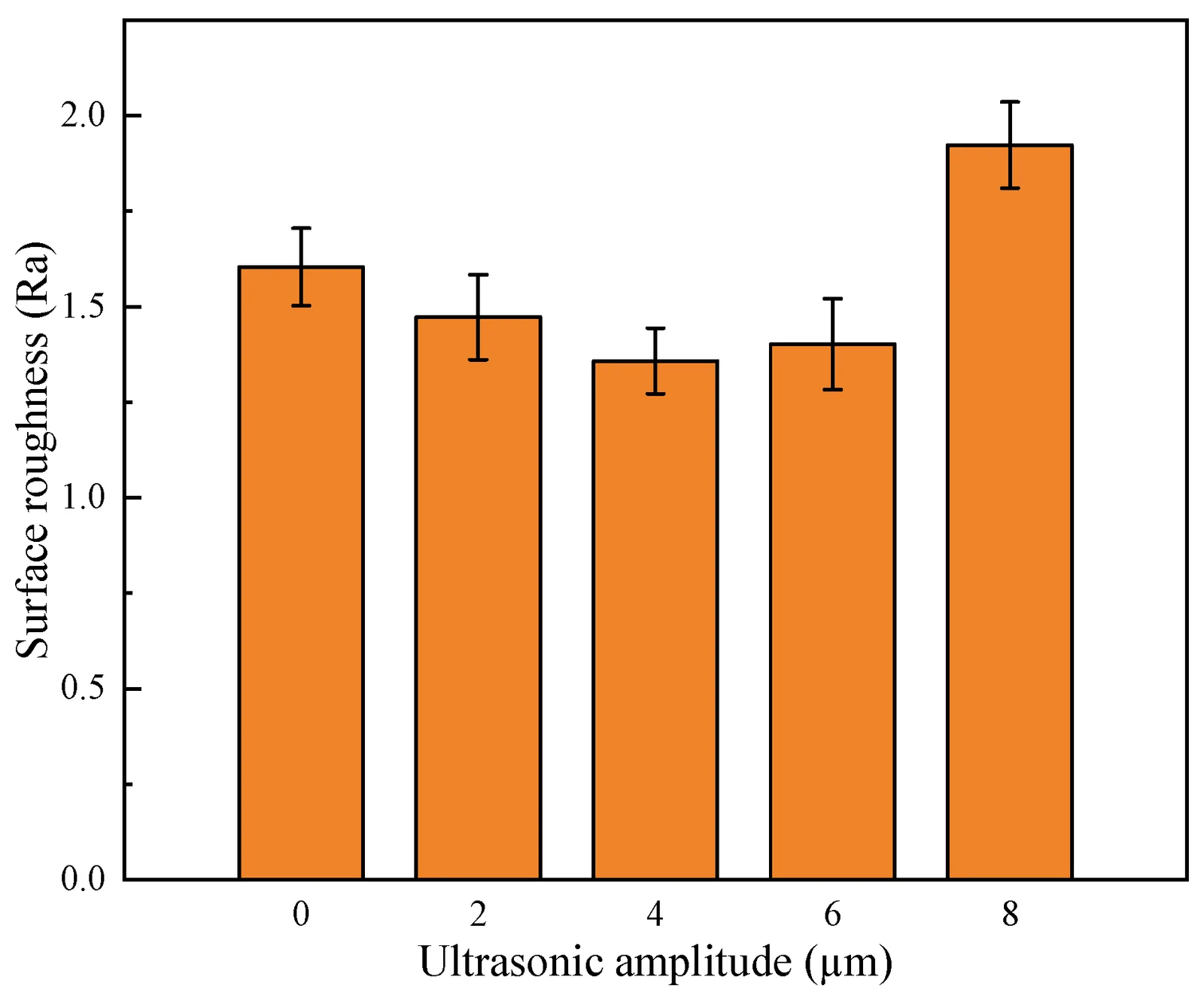
Effect of ultrasonic amplitude on the surface roughness of green ZrO_2_ ceramics under CT and UEVC.

Figure 10 shows the effect of ultrasonic amplitude on the surface roughness of green ZrO_2_ ceramics. The results are consistent with the surface morphology presented in Figure 9. The lowest surface roughness within the tested range was obtained at an ultrasonic amplitude of 4 µm.

### 3.3. Cutting Force

#### 3.3.1. Effect of Depth of Cut on Tangential Forces

**Figure 11:**
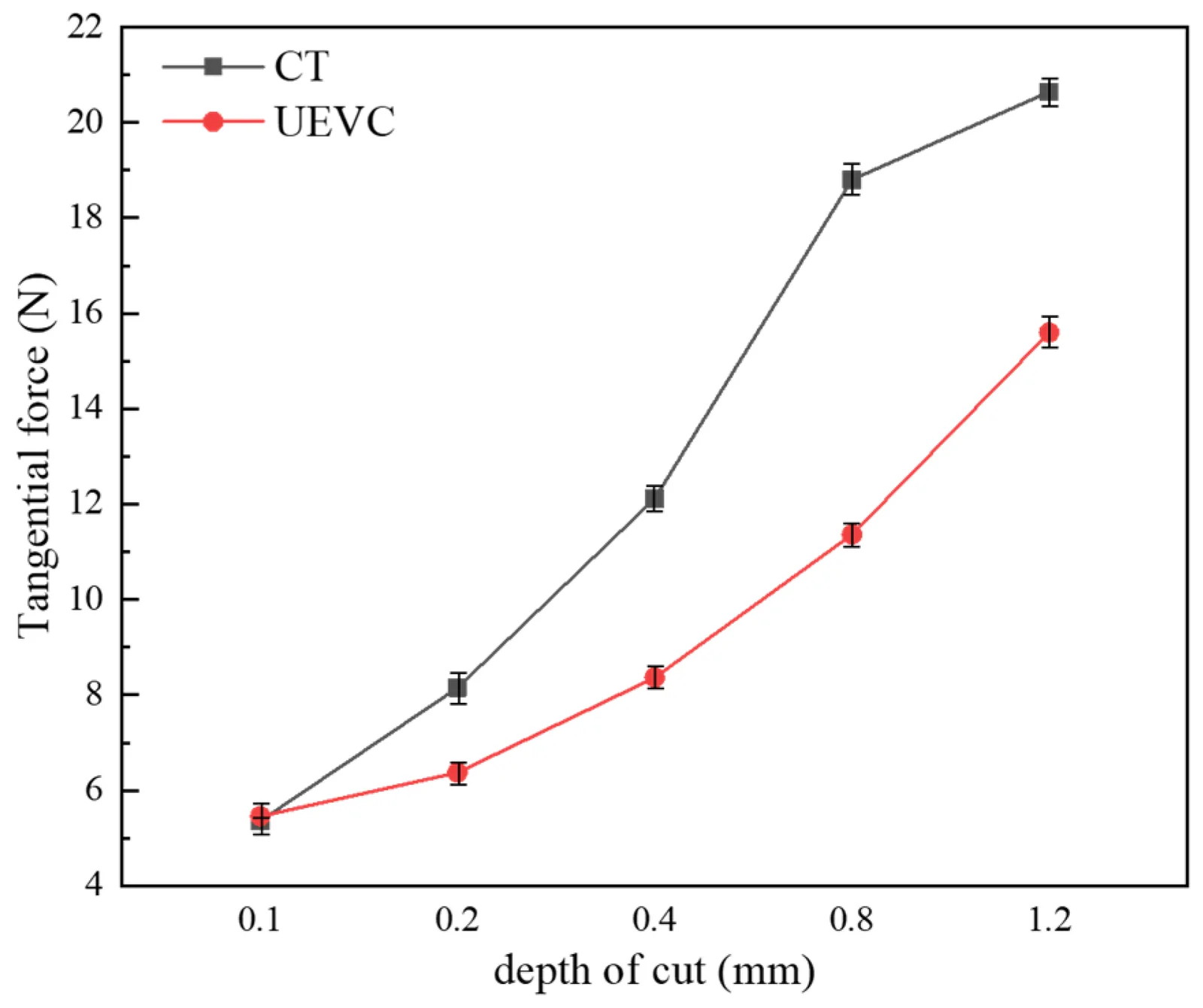
Effect of depth of cut on tangential force under CT and UEVC.

Figure 11 shows the tangential cutting forces under CT and UEVC at different depths of cut. The results indicate that ultrasonic vibration effectively reduces the tangential force during the machining of green ZrO_2_ ceramics. At depths of cut of 0.2, 0.4, 0.8, and 1.2 mm, the tangential forces under UEVC are 6.37 N, 8.36 N, 11.36 N, and 15.60 N, respectively. These values are 21.82%, 30.99%, 39.55%, and 24.44% lower than those under CT. At small depths of cut, the reduction in tangential force achieved by ultrasonic vibration is limited. This is because the contact area between the tool and the workpiece is small. At larger depths of cut, the reduction becomes very pronounced, which is attributed to the intermittent cutting between the tool and the workpiece.

#### 3.3.2. Effect of Cutting Speed on Tangential Forces

**Figure 12:**
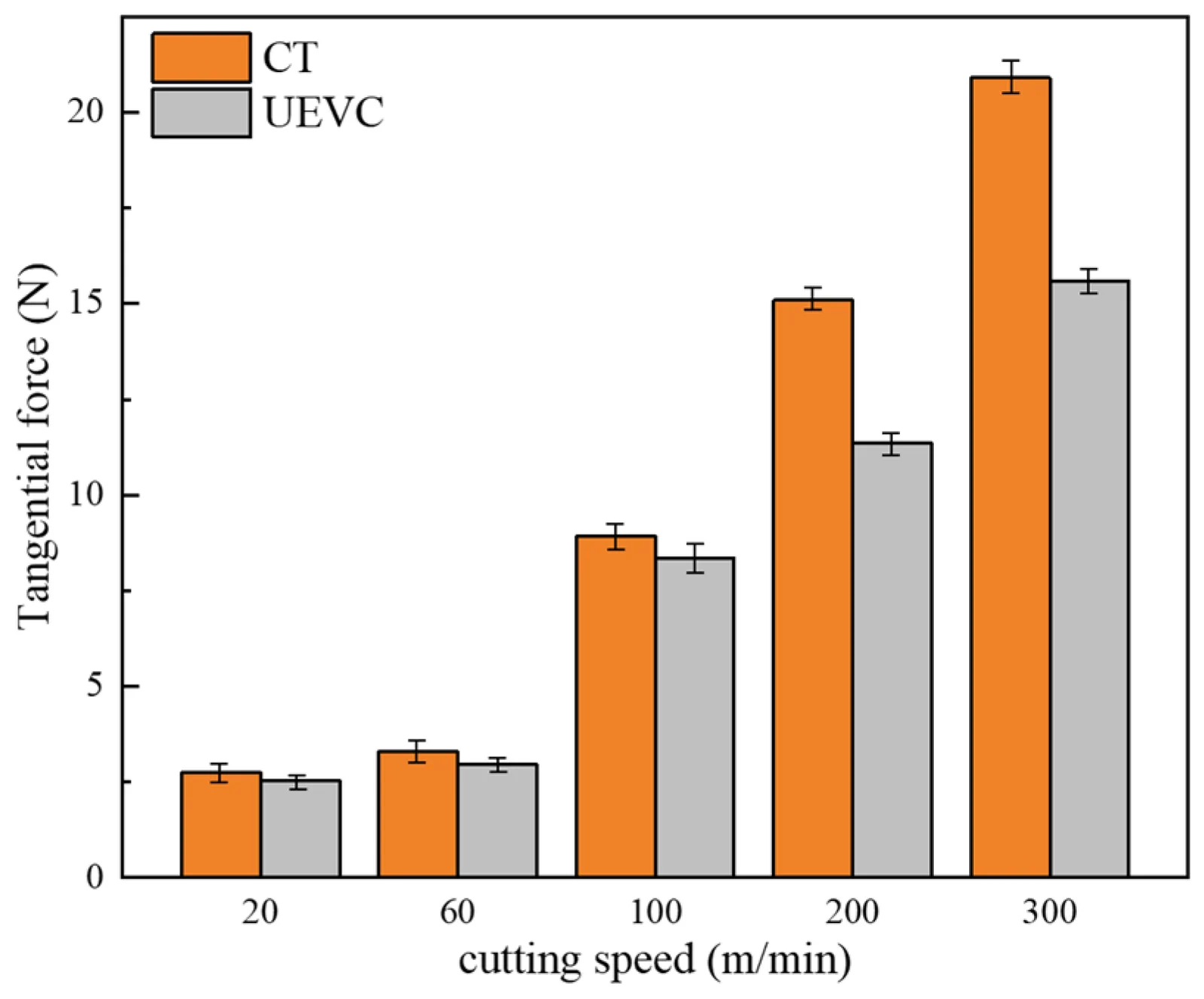
Effect of cutting speed on tangential force under CT and UEVC.

Figure 12 shows the tangential forces under CT and UEVC at different cutting speeds. The tangential force increases with the cutting speed under both cutting conditions. At cutting speeds of 20, 60, and 100 m/min, the tangential forces under CT are 2.74 N, 3.30 N, and 8.96 N, respectively. The corresponding forces under UEVC are 2.54 N, 2.956 N, and 8.363 N. The reductions are relatively small, about 7%, 10.4%, and 6.3%, respectively. Given the small magnitude of these differences and the potential variability in cutting tests, these observations are interpreted as descriptive trends rather than statistically confirmed effects. When the cutting speed increases to 200 and 300 m/min, the tangential forces under CT reach about 15.1 N and 20.9 N. Under the same conditions, the forces under UEVC are 11.363 N and 15.593 N, with much greater reductions of about 24.8% and 25.4%. Overall, UEVC is more effective in reducing the cutting force at high cutting speeds.

#### 3.3.3. Effect of Amplitude on Tangential Forces

**Figure 13:**
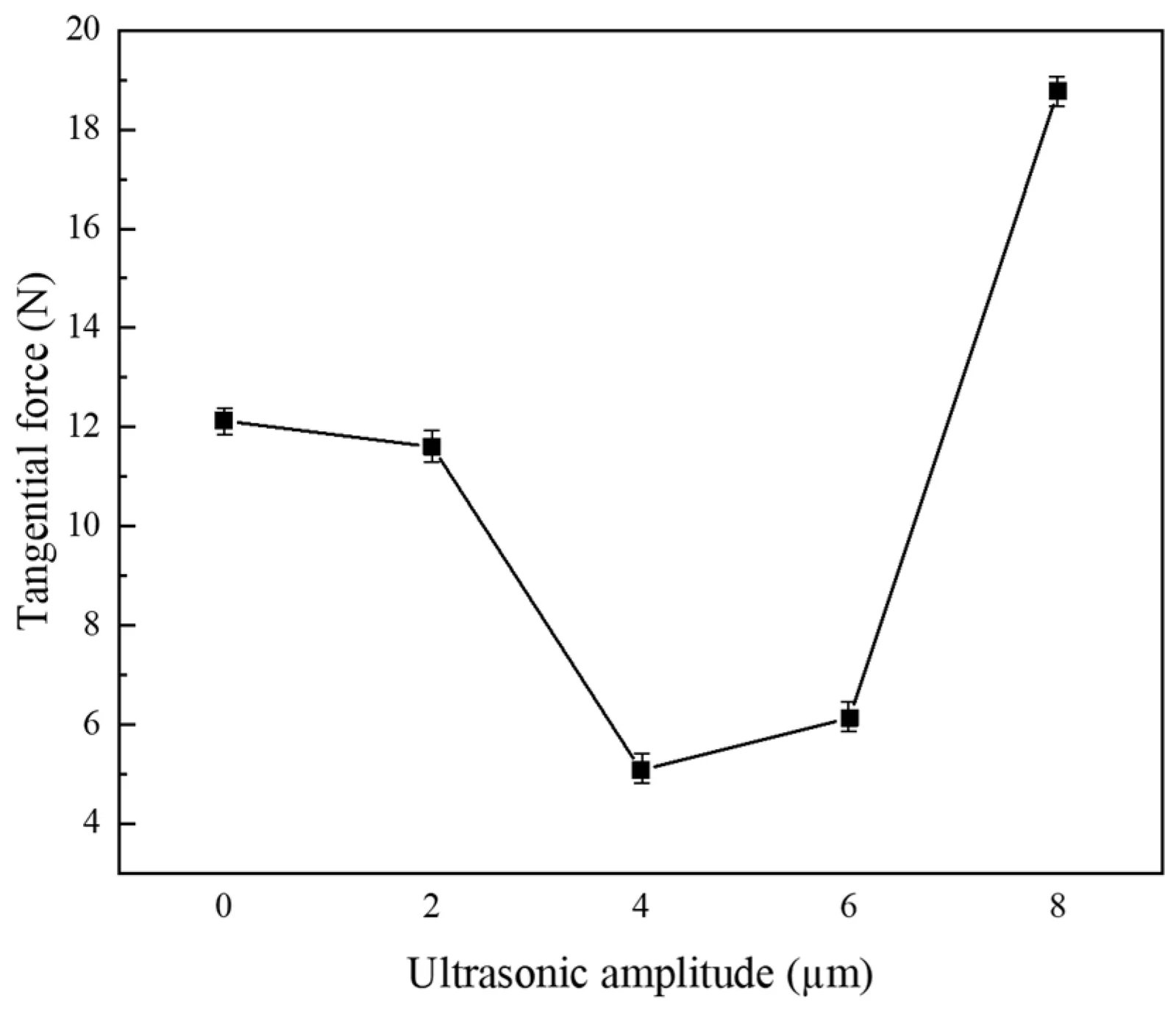
Effect of ultrasonic amplitude on tangential force.

Figure 13 shows the tangential forces during UEVC of green ZrO_2_ ceramics at different ultrasonic amplitudes. The smallest cutting force, 5.073 N, is obtained at an amplitude of 4 µm. Compared with CT, amplitudes ranging from 2 to 6 µm reduce the tangential force. The average tangential force decreases slowly as the amplitude increases from 0 to 2 µm, and it decreases markedly from 2 to 4 μm. The forces at 4 µm and 6 µm show little difference, but the reduction rates are the highest. These rates reach 58.14% and 49.42%, respectively, compared with CT. However, when the amplitude exceeds 6 µm, a large increase in tangential force is observed. This parameter is therefore unsuitable for machining green ceramics. Large cutting forces contribute to the deterioration of surface quality, as shown in Figure 9 and Figure 10.

### 3.4. Surface Compaction Layer

**Figure 14:**
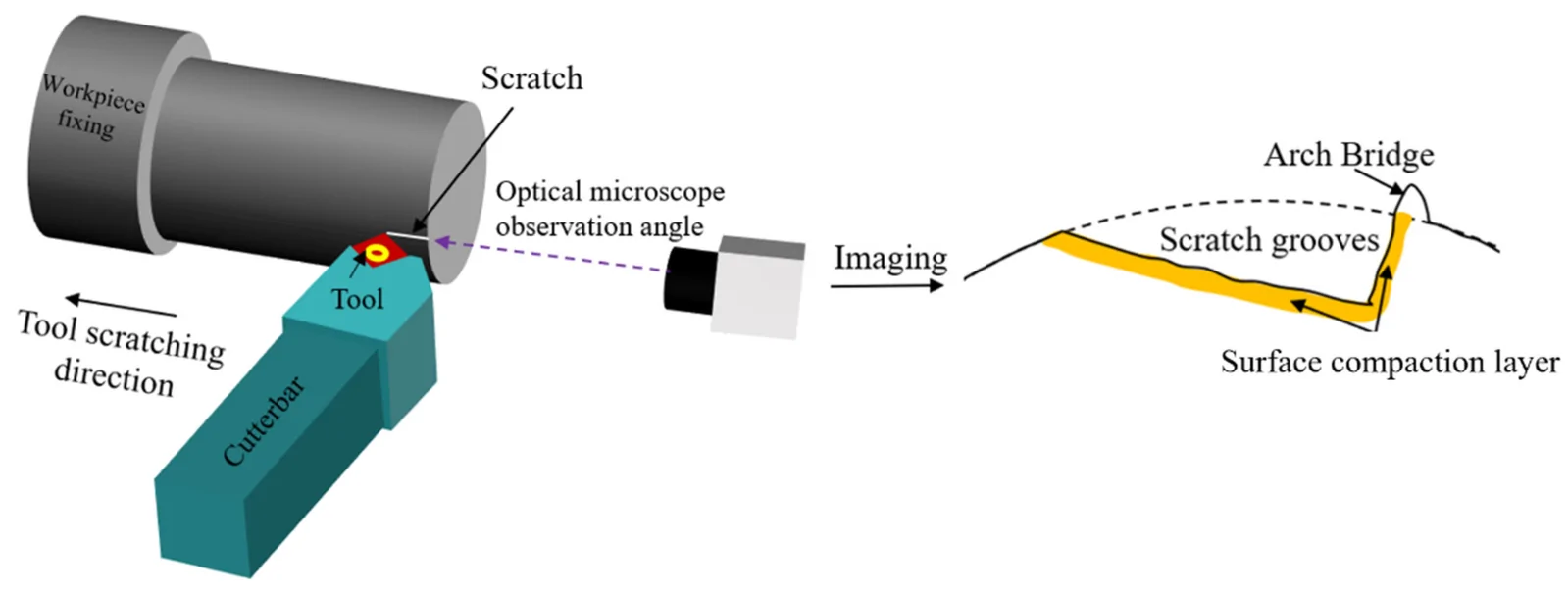
Schematic of scratch test for surface compaction layer of green ZrO_2_ ceramics.

**Figure 15:**
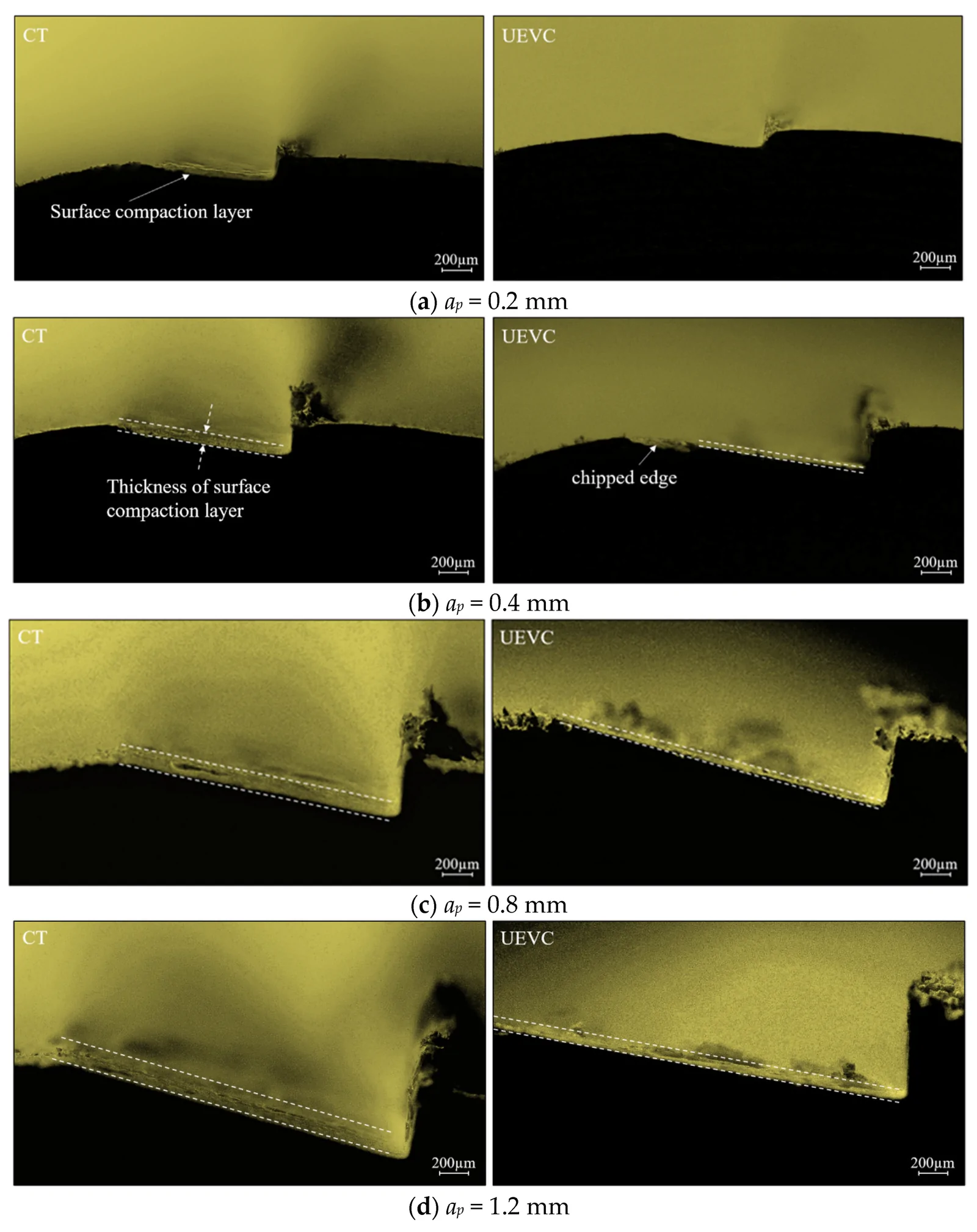
Cross-sectional morphology of scratch grooves at different cutting depths.

Surface compaction layers form on the machined green ZrO_2_ ceramics due to tool extrusion during cutting. The compaction layer is characterized by scratch testing, as illustrated in Figure 14. During each test, the workpiece is clamped on the lathe with the spindle stationary, and the cutting tool moves linearly over a distance of 20 mm in the axial direction at a constant translation speed of 100 m/min. The nominal scratch depth is set by adjusting the radial position of the tool with the lathe cross-slide. Nominal scratch depths of 0.2, 0.4, 0.8, and 1.2 mm are investigated. The scratch tests use the same type of SCMT09T304 cemented-carbide insert as the cutting experiments; the insert has a nose radius of 0.4 mm. To evaluate the compaction layer, the cross-sectional morphology of the scratch grooves at different depths of cut is observed by optical microscopy, as presented in Figure 15. The location of the surface compaction layer and its thickness are indicated in Figure 15a and Figure 15b, respectively. As shown in Figure 15, a visible morphological transition occurs between the compressed and plastically deformed region immediately beneath the groove surface and the unaffected material away from the groove. This morphological transition is used to define the lower boundary of the surface compaction layer. The layer thickness is measured perpendicular to the groove surface from the groove surface to this boundary. The scratch tests are conducted under the experimental conditions listed in Table 6. For each groove, the compaction-layer thickness is measured at five positions to assess variations in layer thickness along the groove. These five measurements are not treated as independent experimental replicates; their mean and standard deviation are reported for each groove. According to Figure 14 and Figure 15, material removal is dominated by plowing and plastic extrusion. An arch bridge forms on one side of the groove due to extrusion and plastic deformation of the material.

Figure 15 shows that a surface compaction layer forms along the scratch groove and becomes thicker as the depth of cut increases. An arch-shaped ridge is also observed on one side of the groove due to plastic deformation. At the same depth of cut, UEVC produces a noticeably thinner surface compaction layer than CT.

**Figure 16:**
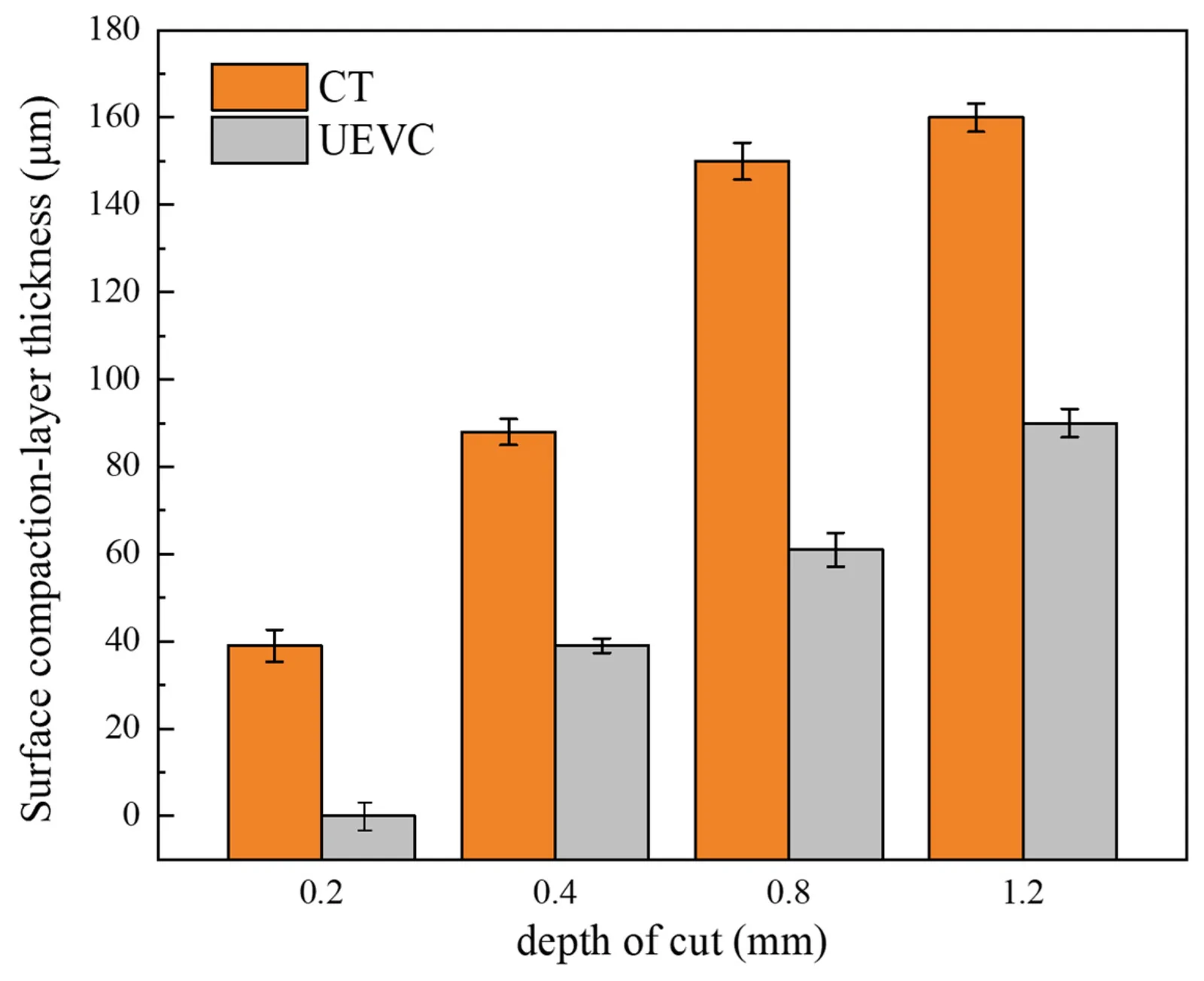
Effect of depth of cut on surface compaction layer thickness.

Figure 16 shows the variation in the surface compaction layer thickness with the depth of cut for green ZrO_2_ ceramics. Under CT, the compaction layer thickness increases rapidly as the depth of cut rises from 0.2 mm to 0.8 mm. In contrast, the thickness under UEVC at a depth of cut of 0.2 mm is nearly 0 µm. At depths of cut of 0.4, 0.8, and 1.2 mm, the compaction layer thicknesses under UEVC are 39 µm, 61 µm, and 90 µm, respectively. These values represent reductions of 55.68%, 59.33%, and 43.75% compared with CT. These results show that UEVC reduces the surface compaction-layer thickness during the machining of green ZrO_2_ ceramics.

## 4. Discussion

### 4.1. Transformation of Material Removal Mode

**Figure 17:**
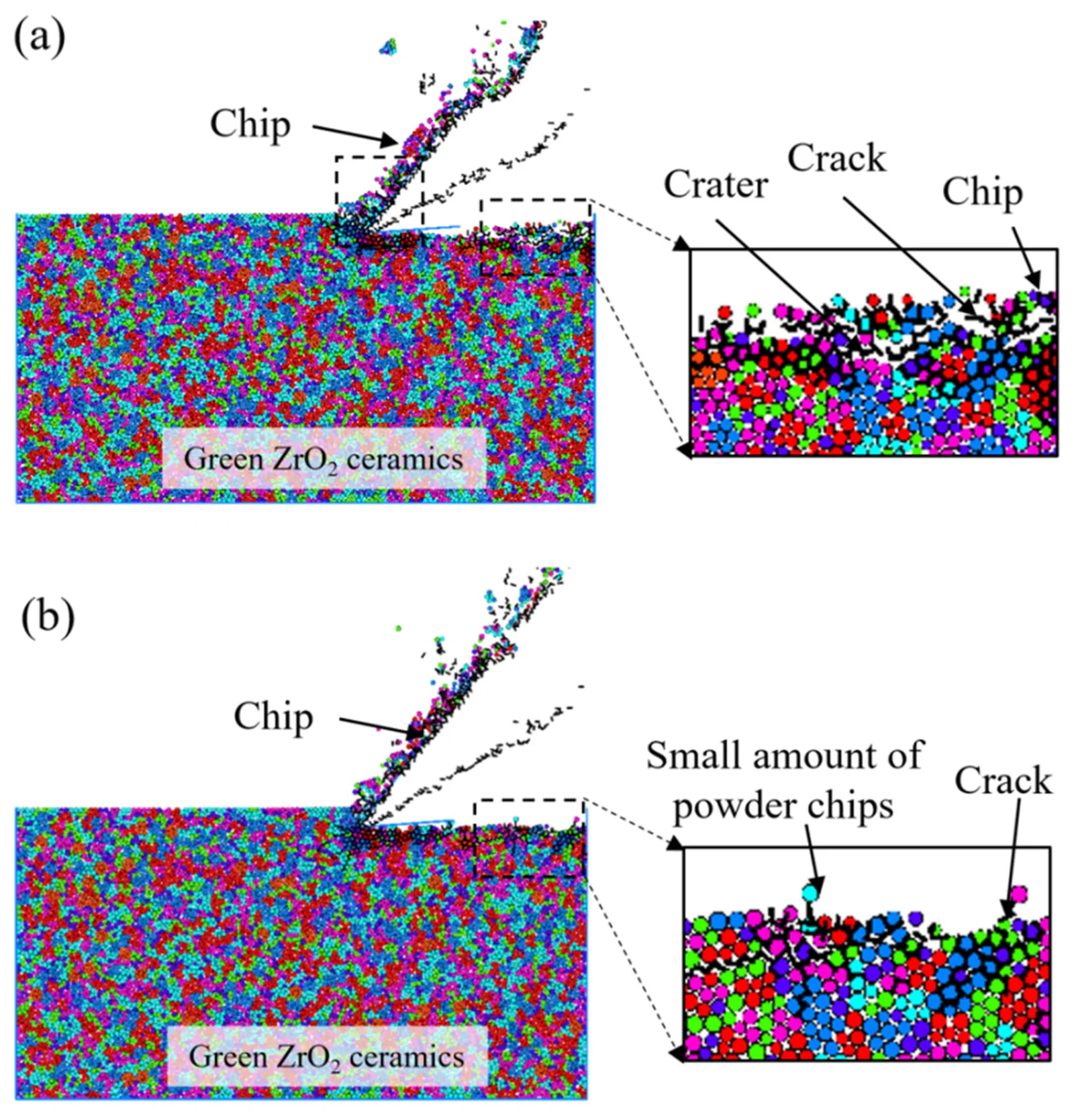
Bonded particle model (BPM)-simulated surface topography and chip morphology of green ZrO_2_ ceramics after machining: (**a**) CT, (**b**) UEVC.

The discrete element simulations in Figure 17 reveal a fundamental change in the material removal mode of green ZrO_2_ ceramics under UEVC. CT (Figure 17a) generates block-shaped chips. These large chips are difficult to evacuate and tend to scratch both the workpiece and the cutting tool, leading to severe surface damage and accelerated tool wear.

Compared with CT, UEVC (Figure 17b) produces a flatter machined surface and powder-shaped chips. Based on the BPM results, the transition from block-shaped to powder-shaped chips is interpreted as a shift from predominantly intergranular fracture under CT to predominantly transgranular fracture under UEVC. The high-frequency elliptical vibration imposes cyclic impact loads on the workpiece, generating stress waves that propagate through the particles, thus causing fracture to occur through the grains rather than along the weak particle boundaries. The powder-shaped chips are easily flushed away from the cutting zone, minimizing secondary damage.

### 4.2. Improvement of Surface Quality and Tool Wear by UEVC

**Figure 18:**
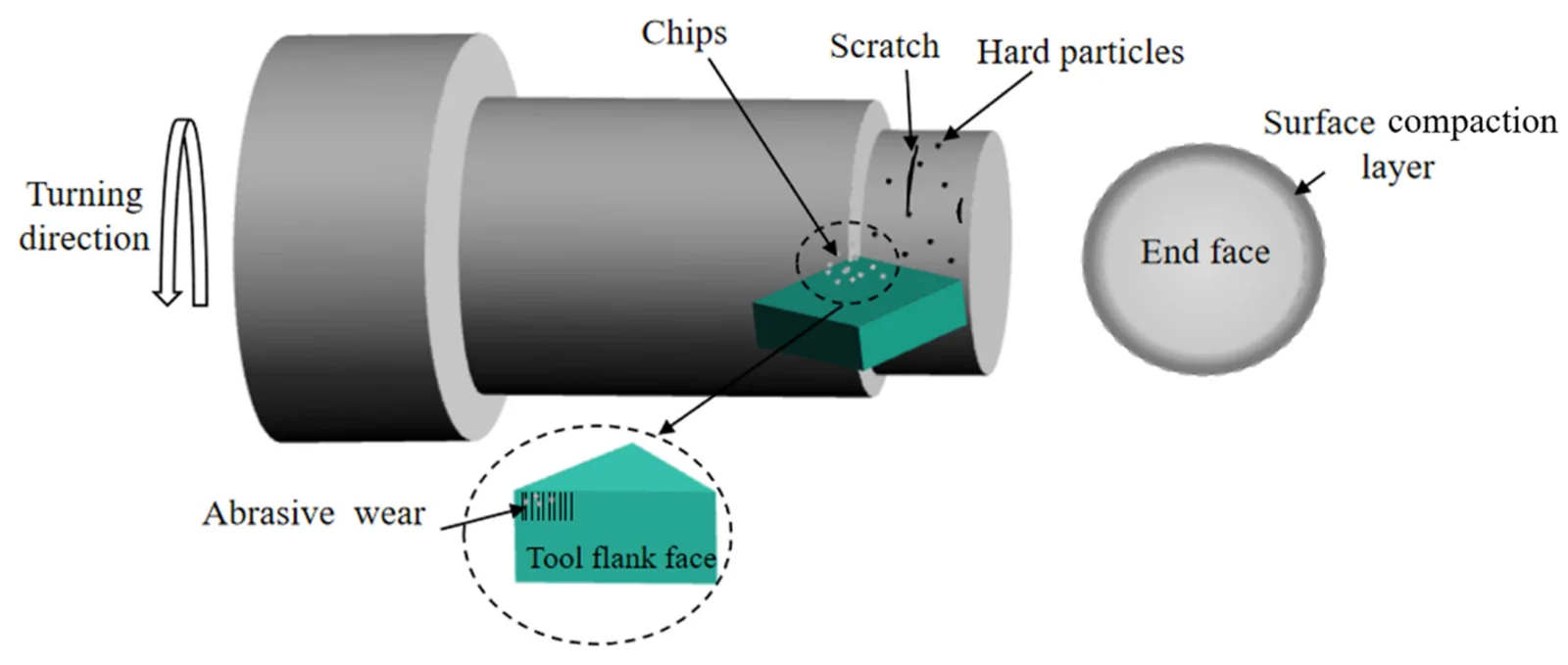
Cutting diagram of green ZrO_2_ ceramics.

In UEVC, the transformation of the material removal mode not only improves the machined surface quality but also reduces tool wear. As shown in Figure 18, hard particles on the workpiece surface continuously scratch the tool flank face, leading to abrasive wear and edge chipping. These abrasive wear features are consistent with the rough surface morphology and deep plowing furrows observed under CT in Figure 5, Figure 6, Figure 7, Figure 8, Figure 9 and Figure 10.

Figure 6 and Figure 8 consistently show lower surface roughness values under UEVC across different cutting parameters. Figure 4b also shows less flank wear compared with CT. The reduction in cutting force under UEVC (Figure 13) is accompanied by less plastic plowing and material spalling on the machined surface. For conditions where the differences between CT and UEVC are modest, such as the cutting force reductions of 6–10% at cutting speeds of 20–100 m/min and the surface roughness improvement of approximately 6% at a cutting speed of 20 m/min, these observations are presented as descriptive trends rather than as statistically confirmed effects. Consequently, under all depths of cut, cutting speeds, and amplitudes, UEVC surfaces exhibit fewer craters and shallower tool marks (Figure 5, Figure 7 and Figure 9). These advantages highlight that UEVC achieves greater improvements than CT, especially at larger depths of cut (Figure 6), higher cutting speeds (Figure 8), and the optimal amplitude of 4 µm (Figure 10), where the improvement in surface quality and the reduction in tool wear are more pronounced.

### 4.3. Low-Damage Mechanism of UEVC for Green ZrO_2_ Ceramics

**Figure 19:**
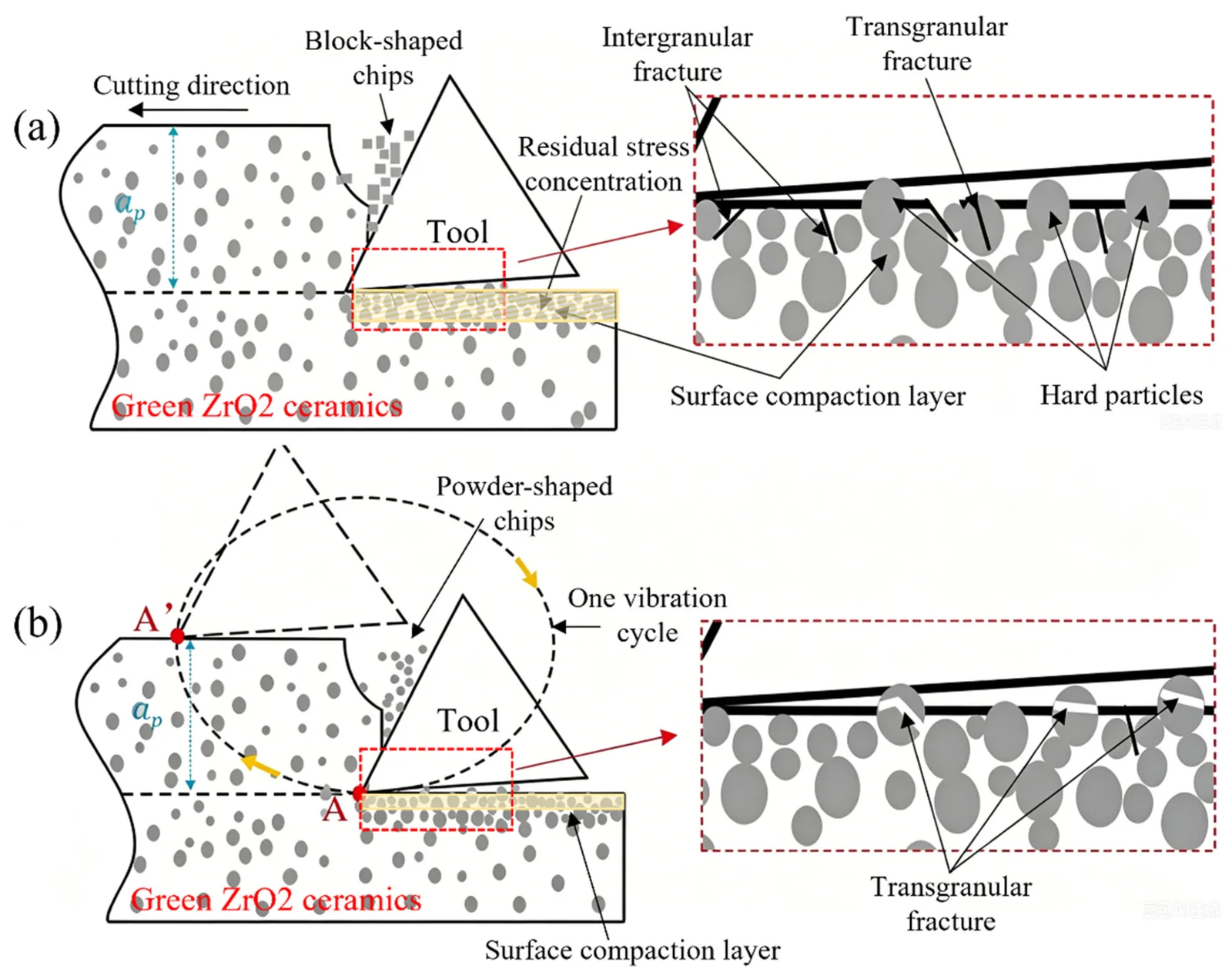
Damage mechanisms of green ZrO_2_ ceramics under different machining methods: (**a**) CT, (**b**) UEVC.

The low-damage machining mechanism of green ZrO_2_ ceramics under UEVC is mainly attributed to the intermittent cutting induced by high-frequency elliptical vibration. Under CT, continuous contact between the tool and workpiece is shown in Figure 19a. Large block-shaped chips remain in the cutting zone and scratch the machined surface, aggravating surface roughness. In contrast, the elliptical vibration trajectory of UEVC causes periodic separation between the tool and workpiece (Figure 19b). During one vibration cycle, the tool contacts the workpiece while moving from point A to point A′. After passing point A′, the tool separates from the workpiece. During this separation period, the tool flank face does not contact the machined surface. According to the BPM-supported mechanistic interpretation, periodic tool–workpiece separation and the associated stress waves promote a shift from predominantly intergranular to predominantly transgranular fracture, resulting in powder-shaped chips. These chips are more readily evacuated, thereby reducing secondary scratching of both the machined surface and the tool flank face.

**Figure 20:**
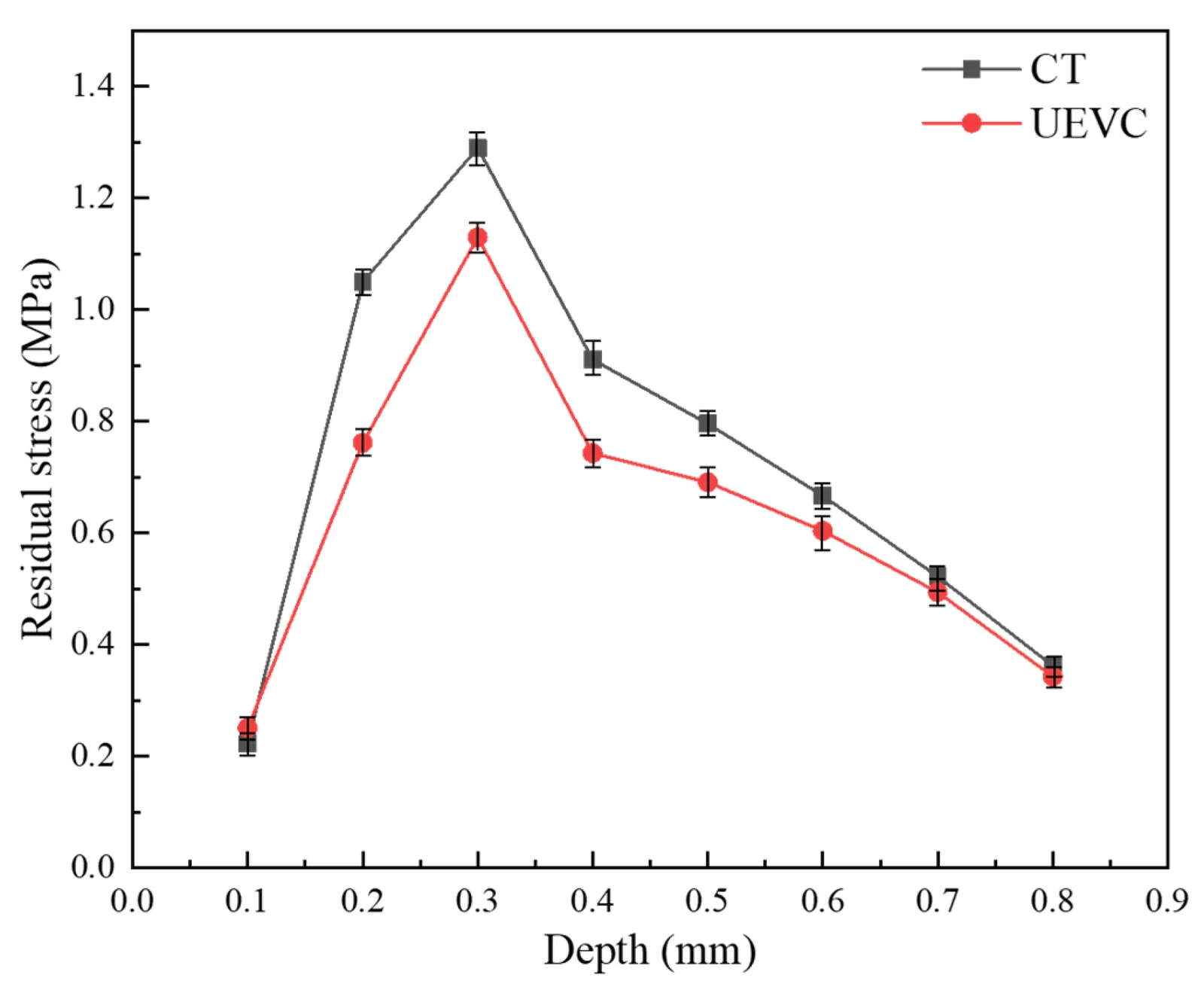
Numerically predicted mean subsurface residual stress magnitudes under CT and UEVC.

Furthermore, UEVC reduces the cutting force, suppresses the formation of a surface compaction layer, and lowers subsurface residual stresses as well as tool flank wear, thereby improving the machined surface quality (Figure 5, Figure 6, Figure 7, Figure 8, Figure 9 and Figure 10). Under CT, the cross-sectional morphology of scratch grooves reveals a distinct compaction layer whose thickness increases rapidly with the depth of cut (Figure 15). In contrast, UEVC produces a much thinner compaction layer at all depths of cut (Figure 15 and Figure 16), with a maximum reduction of 59.33% at a depth of 0.8 mm (Figure 16). The reduction in surface compaction-layer thickness is accompanied by lower subsurface residual stress magnitudes in the PFC2D simulations under UEVC than under CT (Figure 20).

The BPM simulations indicate that subsurface damage under UEVC is primarily confined to the near-surface region (Figure 17). The subsurface residual stress profiles shown in Figure 20 were obtained from the PFC2D simulations using the method described in Section 2.3. UEVC produces a thinner surface compaction layer (Figure 16) and lower simulated subsurface residual stresses (Figure 20). Compared with CT, UEVC achieves better surface quality and reduced tool wear.

The PFC2D model is limited to a two-dimensional cross-section and cannot fully capture the three-dimensional tool geometry, out-of-plane material flow, or three-dimensional chip formation. Therefore, the simulation results are used primarily for qualitative comparisons between CT and UEVC.

## 5. Conclusions

Ultrasonic vibration is applied to the cutting of green ZrO_2_ ceramics to study the surface damage and tool wear under CT and UEVC with different cutting parameters, and the following conclusions are drawn:

1. Compared with CT, UEVC reduces cutting forces and improves machined surface quality. It also suppresses the compaction layer and reduces tool wear.
2. Under certain cutting parameters, UEVC reduces surface roughness by up to 23.2%, the compaction layer thickness by 59.33%, the tangential cutting force by 58.14%, and the average flank wear by 11.5%.
3. The low-damage mechanisms of UEVC for green ZrO_2_ ceramics can be attributed to the following factors. UEVC generates powder-shaped chips rather than the block-shaped chips produced by CT, thereby facilitating chip evacuation and reducing secondary damage from chip scratching. The lower cutting force under UEVC is accompanied by less abrasion by hard particles and fewer craters on the machined surface. Moreover, UEVC reduces the thickness of the machining-induced compaction layer. The PFC2D simulations predict lower subsurface residual stress magnitudes under UEVC than under CT.

## Figures and Tables

**Table 1:** Chemical composition of ZrO_2_ powders [23].

Oxides	ZrO_2_	Fe_2_O_3_	SiO_2_	TiO_2_	Al_2_O_3_	Na_2_O	CaO
wt%	99.9	≤0.003	≤0.03	≤0.002	≤0.002	≤0.001	≤0.002

**Table 2:** Geometric parameters of the cutting tools.

Tool Geometry Characteristics	Characteristic Values
Tool thickness(mm)	3.97
Cutting-edge length (mm)	9
Rake angle (°)	−5
Clearance angle (°)	7
Included angle (°)	90
Nose radius (mm)	0.4
Cutting-edge preparation	Edge-honed

**Table 3:** Ultrasonic vibration system: main technical parameters [24].

Parameter	Numerical Value
Operating voltage (V)	220
Operating power (W)	1000
Vibration frequency (kHz)	37.5
Maximum amplitude (µm)	10

**Table 4:** Experimental conditions for CT and UEVC tests [24].

No.	Cutting Depth (mm)	Cutting Speed (m/min)	Amplitude (µm)	Cutting Method
1	0.1	60	0/4	CT/UEVC
2	0.2	60	0/4	CT/UEVC
3	0.4	60	0/4	CT/UEVC
4	0.8	60	0/4	CT/UEVC
5	1.2	60	0/4	CT/UEVC
6	0.4	100	0/4	CT/UEVC
7	0.4	200	0/4	CT/UEVC
8	0.4	300	0/4	CT/UEVC
9	0.4	60	2	UEVC
10	0.4	60	4	UEVC
11	0.4	60	6	UEVC
12	0.4	60	8	UEVC

**Table 5:** Calibrated parameters of the linear parallel-bond model.

Parameter	Intra-Cluster Contacts	Inter-Cluster Contacts
Linear normal-to-shear stiffness ratio	1.0	0.6
Parallel-bond normal-to-shear stiffness ratio	2.0	1.2
Parallel-bond friction angle (°)	0.5	0.3
Contact friction coefficient	0.6	0.36
Normal critical damping ratio	0.4	0.24
Parallel-bond effective modulus (MPa)	130	78
Parallel-bond tensile strength (MPa)	2.0	1.2
Parallel-bond cohesion (MPa)	1.9	1.14

**Table 6:** Experimental conditions for the scratch tests on green ZrO_2_ ceramic compacts.

No.	Depth of Cut (mm)	Amplitude (µm)	Cutting Method	Scratching Speed (m/min)
1	0.2	0/4	CT/UEVC	100
2	0.4	0/4	CT/UEVC	100
3	0.8	0/4	CT/UEVC	100
4	1.2	0/4	CT/UEVC	100

## Data Availability

The original contributions presented in this study are included in the article. Further inquiries can be directed to the corresponding author.
